# Beneath the surface: revealing deep-tissue blood flow in human subjects with massively parallelized diffuse correlation spectroscopy

**DOI:** 10.1117/1.NPh.12.2.025007

**Published:** 2025-04-09

**Authors:** Lucas Kreiss, Melissa Wu, Michael Wayne, Shiqi Xu, Paul McKee, Derrick Dwamena, Kanghyun Kim, Kyung Chul Lee, Kyle R. Cowdrick, Wenhui Liu, Arin Ülkü, Mark Harfouche, Xi Yang, Clare Cook, Seung Ah Lee, Erin Buckley, Claudio Bruschini, Edoardo Charbon, Scott Huettel, Roarke Horstmeyer

**Affiliations:** a Duke University, Department of Biomedical Engineering, Durham, North Carolina, United States; b École polytechnique fédérale de Lausanne (EPFL), Advanced Quantum Architecture Laboratory, Neuchatel, Switzerland; c Duke University, Department of Psychology and Neuroscience, Durham, North Carolina, United States; d Seoul National University, Department of Mechanical Engineering, Seoul, Republic of Korea; e Seoul National University, School of Mechanical & Aerospace Engineering/SNU-IAMD, Seoul, Republic of Korea; f Georgia Institute of Technology and Emory University, Wallace H. Coulter Department of Biomedical Engineering, Atlanta, Georgia, United States; g Tsinghua University, Department of Automation, Beijing, China; h Ramona Optics, Inc., Durham, North Carolina, United States

**Keywords:** parallelized diffuse correlation spectroscopy, blood flow, single photon avalanche diodes, cerebral blood flow, diffuse optics, single photon avalanche diodes arrays

## Abstract

**Significance:**

Diffuse correlation spectroscopy (DCS) allows label-free, non-invasive investigation of microvascular dynamics deep within tissue, such as cerebral blood flow (CBF). However, the signal-to-noise ratio (SNR) in DCS limits its effective cerebral sensitivity in adults, in which the depth to the brain, through the scalp and skull, is substantially larger than in infants.

**Aim:**

Therefore, we aim to increase its SNR and, ultimately, its sensitivity to CBF through new DCS techniques.

**Approach:**

We present an *in vivo* demonstration of parallelized DCS (PDCS) to measure cerebral and muscular blood flow in healthy adults. Our setup employs an innovative array with hundreds of thousands single photon avalanche diodes (SPAD) in a 500×500 grid to boost SNR by averaging all independent pixel measurements. We tested this device on different total pixel counts and frame rates. A secondary, smaller array was used for reference measurements from shallower tissue at lower source-detector-separation (SDS).

**Results:**

The new system can measure pulsatile blood flow in cerebral and muscular tissue, at up to 4 cm SDS, while maintaining a similar measurement noise as compared with a previously published 32×32 PDCS system at 1.5 cm SDS. Data from a cohort of 15 adults provide strong experimental evidence for functional CBF activity during a cognitive memory task and allowed analysis of pulse markers. Additional control experiments on muscular blood flow in the forearm with a different technical configuration provide converging evidence for the efficacy of this technique.

**Conclusions:**

Our results outline successful PDCS measurements with large SPAD arrays to enable detect CBF in human adults. The ongoing development of SPAD camera technology is expected to result in larger and faster detectors in the future. In combination with new data processing techniques, tailored for the sparse signal of binary photon detection events in SPADs, this could lead to even greater SNR increase and ultimately greater depth sensitivity of PDCS.

## 1 Introduction

**Fig. 1:**
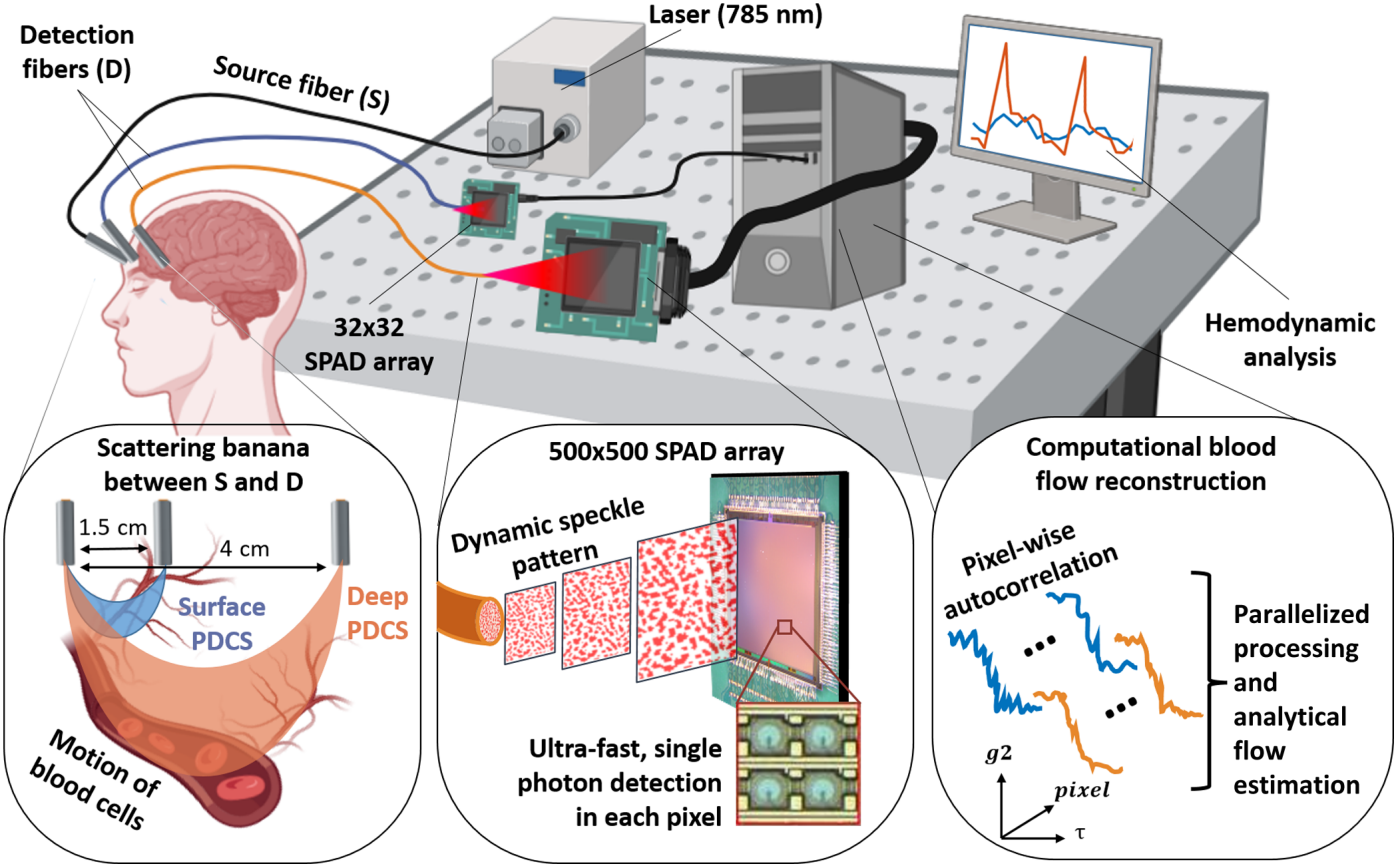
Basic principle and experimental setup of parallelized diffuse correlation spectroscopy (PDCS). A coherent laser source connected to an optical fiber delivers light to the area under investigation, e.g., the forehead for measurement of cerebral blood flow (CBF). Photons emitted by the source fiber (S) scatter diffusely within tissue and a small fraction reach the detection fiber (D). A “banana” shaped probability distribution describes the average optical path between S and D. Photons at D are transmitted to a single-photon avalanche diode (SPAD) array. A short source detector separation (SDS) predominately measures photons scattered in superficial tissue layers (blue). A larger SDS detects more photons that have traveled through deeper tissue layers (orange). Each pixel in the SPAD array measures the temporal dynamics of a single speckle and thereby performs an independent DCS measurement. These individual intensity time traces are then used to compute autocorrelation curves, whose slope (i.e., decorrelation rate) are proportional to blood flow speed within the tissue. PDCS averages autocorrelations across thousands of SPAD array pixels to increase system signal-to-noise ratio.

**Fig. 2:**
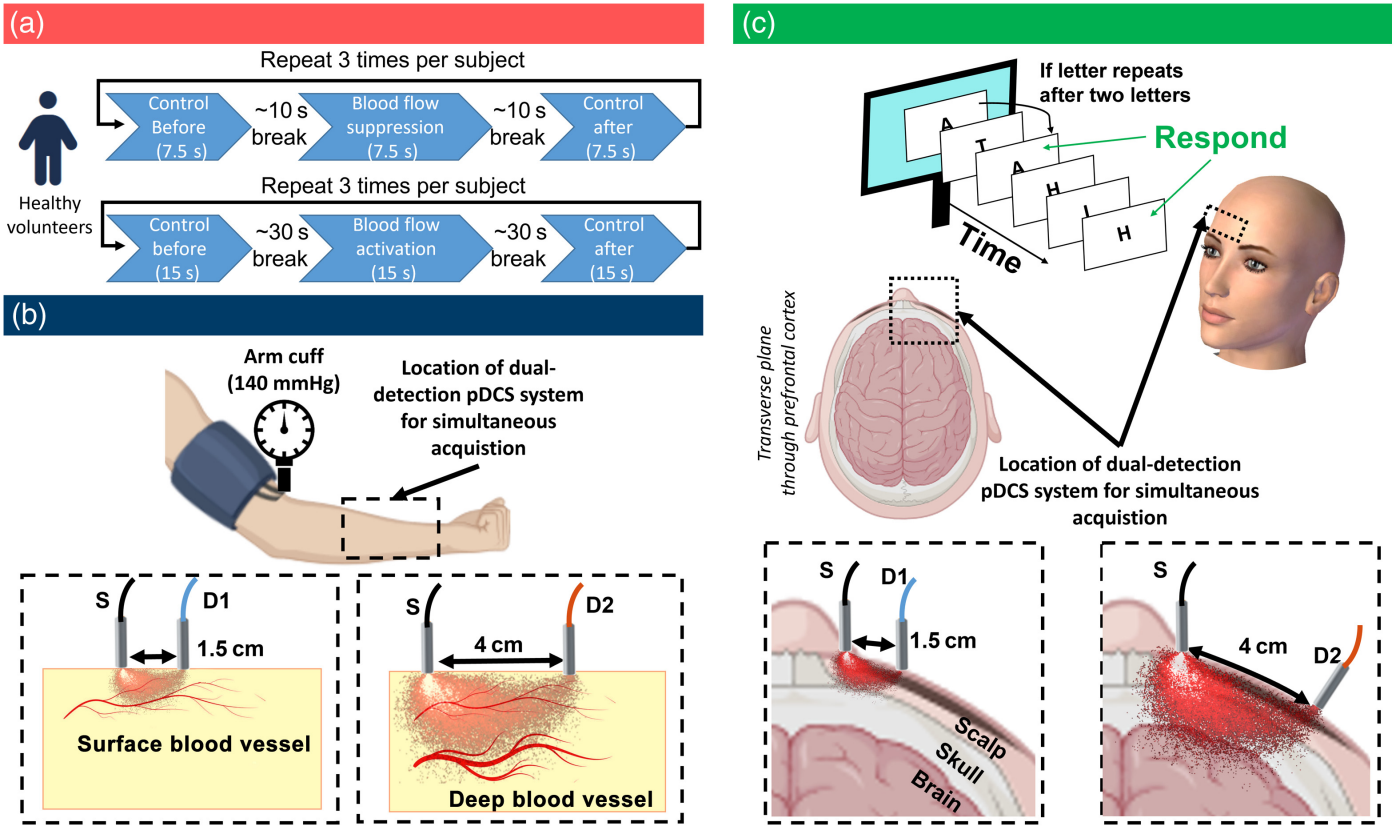
Study design (a) and principle of dual-detection with PDCS (blood flow suppression in forearm via pressure cuff) (b) at the forearm and (c) the forehead (cognitive blood flow activation in prefrontal cortex).

Diffuse correlation spectroscopy (DCS) is a noninvasive, label-free optical tool used to quantify microvascular blood flow in biological tissue. In a traditional DCS setup, a coherent continuous wave laser illuminates a turbid sample, whereas multiply-scattered photons are collected at a known distance to this illumination spot (see Fig. 1). Photons that are emitted by the source (S) are scattered randomly multiple times within the tissue, with a small fraction re-emerging at the surface of the sample at the detector (D). DCS is based on measuring ultra-fast intensity fluctuations of this re-emerging, diffusely scattered light. In biological tissue, these intensity fluctuations arise from the motion of red blood cells (RBC). Analytical models relate these intensity fluctuations to a blood flow index (BFI) in the underlying tissue.^1^^,^^2^ The summation of all individual photon paths between S and D leads to a stochastic scattering profile, with a characteristic “banana-shape,” where larger source detector separations (SDS) lead to greater depth sensitivity^3^ [see Figs. 1, 2(b), and 2(c)]. However, the detected light intensity also falls off exponentially with greater SDS, which reduces the measurement signal to noise ratio (SNR). Thus, experiments are designed to balance maximizing depth sensitivity while ensuring sufficient SNR. Most commonly, DCS has been used at SDS = 2 to 2.7 cm,^4^^–^^8^ relating to a rough estimate of ∼1 to 1.35 cm penetration depth.^9^

This constrained depth sensitivity of conventional DCS systems is especially relevant for measurements of cerebral blood flow (CBF) because the cerebral tissue of interest is beneath the barrier of the scalp and skull. In human adults for instance, the average distance from scalp to brain can be at the order of ∼1 to 1.5 cm (i.e., 1.48±0.28  cm^10^). Thus, there is a need to enhance the signal-to-noise ratio (SNR) and ultimately the depth penetration of traditional DCS to improve sensitivity to CBF. In addition, DCS measurements of CBF are typically characterized by faster intensity fluctuations/faster decorrelation rates^11^ as compared with other tissues, such as the muscle,^12^ which therefore requires faster intensity measurements for CBF. In contrast to that, DCS measurements of muscular blood flow (MBF)^12^ are typically less challenging in terms of SNR, depth sensitivity, and measurement speed, which led to several applications, for instance to measure the effects of exercise^13^ or to investigate pathological conditions, such as peripheral arterial disease.^14^

Several techniques have been developed to address these limitations for CBF measurements and to enhance the depth sensitivity of DCS.^1^^,^^11^^,^^15^^–^^17^ Some approaches use more advanced detection schemes, such as Fourier-domain DCS, which is based on interference and intensity modulation,^18^ or time-domain DCS, which is based on time-of-flight detection.^19^ Other approaches acquire DCS measurements at multiple SDS and employ advanced computational inversion models to estimate BFI, such as multi-layer models (MLM), which can account for different optical properties and exploit the fact that different regions in the autocorrelation curve are sensitive to different depths.^1^^,^^20^ Interferometric DCS (iDCS), for instance, are sensitive to faster speckle variations, which is generally advantageous for deep-tissue DCS.^21^^–^^23^ Functional interferometric diffusing wave spectroscopy (fiDWS) has been reported at 4 cm SDS and 10 Hz sampling rate or even at 5 cm SDS and 0.1 Hz sampling rate.^22^

The concept of measuring spatial contrast of the speckle pattern by complementary metal-oxide-semiconductor (CMOS) cameras has also developed as a promising alternative to DCS.^24^^,^^25^ This concept is known as speckle contrast optical spectroscopy (SCOS),^24^ speckle visibility spectroscopy (SVS),^26^ or diffuse speckle contrast analysis (DSCA)^27^ and can achieve SNR improvement at reduced cost as compared with traditional DCS.^28^ Recently, SCOS was implemented at 3.3 cm SDS and 10 Hz sampling rate by carefully characterizing, selecting, and optimizing the CMOS camera^29^ and Kim et al. showed SCOS at 4 cm SDS and 46 Hz sampling rate.^28^ Similar to iDCS, interferometric systems have also been shown for SVS (iSVS), for instance at a BFI sampling rate of 100 Hz and SDS of 1.5 cm^30^ or even at a sampling rate of 125 Hz and SDS of 3 cm.^31^ These speckle contrast and interferometric methods show significant promise for deep-tissue BFI measurement and can potentially offer complimentary or extended benefits to the technique used in this work.

A rather straight-forward method to increase the SNR of DCS is by simultaneously measuring the temporal fluctuations of multiple independent speckles. In 2007, Dietsche et al. used a fiber bundle, an array of detectors and a multichannel autocorrelator to show DCS at 2.9 cm SDS from a single subject.^32^ A similar concept has been used for “multi-speckle DCS”^33^ or “multiplexed DCS” with an 5×5 array.^34^ This concept of parallelization has also made its way into interferometric setups, such as the “parallel, interferometric diffusing wave spectroscopy.”^21^ These systems measure ∼20,^21^ 25,^34^ 96,^22^ or even up to 980^35^ speckles in parallel, and by averaging all M independent autocorrelation curves, the SNR can consequently be increased by a factor of $\sqrt{M}$ compared with a single pixel. A combined strategy of using a longer wavelength of 1064 nm, an interferometric setup, and multi-speckle detection by four superconducting nanowire single-photon detectors (SNSPD) was deployed by Robinson et al. to measure cerebral blood flow at 100 Hz sampling rate and a SDS of 3.5 cm.^35^

The recent development of larger SPAD arrays with many hundreds and thousands of individual SPAD pixels is fueling this ongoing trend of parallelization and multi-speckle detection. For instance, Sie et al.^36^ as well as Liu et al.^37^ used a 32×32 SPAD array and averaged all 1024 pixels to boost the SNR 32-× compared with a single pixel measurement, terming this technique multispeckle DCS^36^ or parallelized DCS (PDCS).^37^ In this work, we will use the later term PDCS from here on. A similar PDCS setup with a 32×32 SPAD array was then used to classify spatiotemporal-decorrelating patterns deep beneath turbid media^38^ and to exploit such patterns to reconstruct images of flow patterns.^39^ However, it should be noted that the effective SNR-gain in PDCS with SPAD arrays over conventional DCS with individual SPADs is usually below the theoretical factor of $\sqrt{M_{\text{pixel}}}$ because each pixel in the array has a lower photon detection efficiency (PDE) as conventional, single SPADs (e.g., PDE ∼15% at 785 nm in arrays against up to 70% in single SPADs).^16^ Most recently, a large-format SPAD camera with 500×500  pixels has been demonstrated.^40^ Compared with the 1024 pixels in our previous PDCS systems,^37^^–^^39^ this new SPAD array has a total of 250,000 pixels and therefore offers a massively increased potential for parallelized DCS measurements. Optical phantom experiments already showed that this new SPAD array can be used for PDCS experiments, following the same $\sqrt{M_{\mathrm{pixel}}}$ gain in SNR, boosting it by a factor of up to 473 compared with a single SPAD pixel in the array.^41^ Despite this very promising technological advance, SPAD cameras of this massive size have not yet been utilized for *in vivo* experiments to study blood flow in human subjects.

Although the promise of these new DCS technologies for SNR gain is evident, most of these proof-of-concept studies were only carried out on optical phantoms^41^ or on a relatively low number of human subjects (n), e.g., n=7,^8^ n=4,^22^ n=1,^32^ n=3,^33^ n=5,^35^ or n=6,^37^ which reduces a statistical significance analysis of blood flow metrics and SNR behavior under real experimental conditions.

In this publication, we demonstrate the first *in vivo* use of the latest 500×500 SPAD camera for massively parallelized DCS to measure MBF at the forearm and CBF at the prefrontal cortex (PFC) in over a dozen adult subjects and in actual cognitive neuroscience experiments. We show that this new PDCS system offers the ability to optically measure pulsatile blood flow at SDS of 4 cm, enabling a sampling rate of 8 to 10 Hz. A dual-detection system with two different PDCS systems—one at short SDS and one at large SDS—further allowed a direct, in-built comparative reference between superficial and deeper tissue layers. We carried out a systematic study with two different experiments, investigating global suppression of MBF to establish a reliable proof-of-concept, as well as localized activation of CBF in brain tissue, to demonstrate measurements during a functional, cognitive task. Furthermore, because the exposure time of the 500×500 SPAD camera scales with the total number of recorded SPAD pixels, these two experiments were also used to explore the trade-off between flow-sensitivity (increases by faster exposure time) and SNR (increases by averaging more pixels) in our PDCS system.

## 2 Materials and Methods

### 2.1 Study Design

Healthy adult subjects (n=24) were recruited for this study. Participants received compensation either in the form of monetary payment or credit for a requirement within one of their courses. Data were collected from two different locations, under two different conditions: (i) general suppression of muscle blood flow (MBF) in the entire forearm and (ii) localized activation of cerebral blood flow (CBF) at the PFC. At the forearm, the test condition was flow suppression via a blood pressure cuff and PDCS data were collected before, during, and afterwards. In the second experiment, subjects participated in a cognitive task (see below) and PDCS were recorded at the forehead, above the PFC before, during, and after this task. The same procedure was repeated up to 3 times for more independent measurements per subject, as displayed in Fig. 2(a). We refer to these independent measurements as “trials.” However, this procedure did not result in exactly three trials per participant due to time constraints per participant. In total, we acquired 47 complete data sets from 15 unique subjects with both detection schemes in the CBF experiment and 33 complete data sets from 11 unique subjects in the MBF experiment. Participants were briefed about all details of the study, signed a consent form, and wore laser safety goggles throughout the entire experiment. The study protocol was approved by the Duke Campus Institutional Review Board (IRB) prior to the experiments (IRB Protocol Number 2022-0093). Demographics of all subjects are shown in the Supplementary Material.

#### 2.1.1 PDCS measurement at the forearm during blood flow suppression

Subjects placed their right forearm on a table, and the optical setup consisting of three fibers was placed on the arm. A standard blood pressure cuff was placed on the same arm of the subject. During the occlusion, the pressure was set to 140 mmHg. Up to three independent trials were recorded and each trial consisted of an initial control measurement without occlusion, one test measurement during occlusion, and a second control measurement seconds after the release of the pressure. Each measurement interval lasted 7.5 s, with a break of about 10 s between two measurements (i.e., between control and occlusion measurement). Complete and paired data from both detectors were available for 33 trials. A pre-defined exclusion criterion for the residual of BFI estimation was applied to these data and resulted in the removal of nine trials (27% exclusion rate, five cases in the PF-32 data at short SDS, six cases in the swissSPAD3 data at long SDS, including two in both cases) as shown in Fig. S3 in the Supplementary Material. This automated removal of extreme outliers followed the pre-registered analysis plan.^42^

#### 2.1.2 PDCS measurement at the prefrontal cortex during cognitive activation

For measurements of CBF, adult subjects placed their head on a chin rest and the three optical fibers were positioned at the forehead at 1 to 2 cm above the right eyebrow. A small monitor was placed at about 50 cm distance at eye level of the subject, and their hands were placed on a keyboard to control the required keys for the cognitive task. The n-back task (n=2) was carried out on a web service provided by PsyToolkit’s experiment library^43^ based on the procedure explained in Refs. 44 and 45. This task is known to cause activation in the PFC, which is responsible for working memory and executive function.^46^ During this task, a series of 25 letters is shown to the participants, usually containing six instances where the same letter is repeated after two letters [see Fig. 2(c)]. If subjects correctly indicated this instance, it was counted as a correct match. Missed instances and false alarms were also counted. Prior to the actual experiment, each subject was instructed in the procedure and the rules of this n-back test. Each subject carried out one practice test, without data acquisition to familiarize themselves with the test. For all subsequent trails with data acquisition, the percentage of correct matches, the percentage of missed matches, and the percentage of false alarms were documented, as shown in the Supplementary Material. Each trial consisted of an initial control measurement, followed by a measurement during the task and, finally, another control measurement [see Fig. 2(a)]. The actual n-back test lasted about 40 to 50 s, and the PDCS measurement was started about 15 to 20 s after the start of the test. Each PDCS measurement lasted 15 s. After the task was completed, subjects were again asked to close their eyes and a second control measurement was recorded. There was a break of about 20 to 30 s between two measurements in the same trial (i.e., between control measurement and task measurement). The same procedure was repeated up to 3 times, to obtain independent trials for each subject. From the total number of 24 subjects that were recruited, complete data from both detectors were available from 18 subjects and 47 trials. Eight of those trials of the cognitive task were excluded based on the pre-defined exclusion criterion for the residual of the BFI reconstruction for a total of 39 trials from 15 subjects (17% exclusion rate, all eight cases in the sSPAD3 data), as described in detail in the Supplementary Material.

### 2.2 Optical Setup

As displayed in Figs. 1, 2(b), and 2(c), we used a dual PDCS system, with one laser source (λ=785  nm, Crystalaser solid state laser) and two individual detection setups. The laser was operated at 100 mW and connected to a large fiber patch cable (Thorlabs M107L, 1500  μm, NA = 0.50). An empty lens tube (SM1, 25.4 mm diameter) with a length of 16 mm was mounted to the laser fiber to act as a spacer between the fiber tip and the subject’s skin. This arrangement ensured a sufficiently large beam diameter at the skin such that the irradiance fell well below the maximal permissible exposure (MPE) limit of $300\;\mathrm{mW}/cm^{2}$. Based on the NA of the laser fiber and the length of the space, one can estimate the radius of the illumination spot as ∼4  mm. The first detection setup consisted of the commercially available 32×32 SPAD camera (PF-32, PhotonForce Ltd., Scotland, UK) that was connected to a 200  μm fiber (Thorlabs M44L, 200  μm, NA = 0.50). The other fiber end was placed on the forehead at 1.5 cm from the source fiber (SDS = 1.5 cm). The second detection setup was based on the novel 500×500 SwissSPAD3 array^40^ connected to a 1500  μm fiber (Thorlabs M107L, 1500  μm, NA = 0.50), which was spaced 4 cm from the source fiber (SDS = 4 cm). As mentioned above, this dual-detection system was selected to allow a direct, in-built reference between superficial blood flow measured at shorter SDS and blood flow that also includes deeper tissue layers, as measured by the larger array at greater SDS. In both setups, the distance between the detection fiber and the sensor (z) was adjusted to match the speckle diameter (d) to the active area of the detector, according to Ref. 47

$$d=\frac{\lambda z}{D},$$ (1)

where λ is the wavelength (785 nm) and D is the fiber diameter. In the case of the setup at SDS = 1.5 cm, the diameter of the active area of each pixel in the PF-32 array is 6.9  μm, resulting in a distance of z=1.76  mm between fiber and detector. In the case of the long source-detector separation (SDS = 4 cm), the setup followed the procedure described by Wayne et al.,^41^ with a distance of z=11.5  mm between fiber and detector because the diameter of the active area of each pixel in the SwissSPAD3 array is 6  μm. In this configuration, each pixel was sampling a single speckle on average. To ensure a correct setup, the speckle patterns at these distances were measured by a camera with 1.85  μm pixel size (acA4024-29um, Basler AG, Ahrensburg, Germany).

With this configuration, we generally quantify the correlation amplitude β to be between 0.15 and 0.3 in all experiments, with a subject-to-subject variance that was generally was greater than the difference between both setups. To account for this variation, we included β as a fitted parameter in our BFI model, within the boundary values of 0 and 0.7 as discussed in the Supplementary Material.

### 2.3 SPAD Arrays

Here, we summarize the main specifications of the two different SPAD arrays (Table 1). For more details please refer to the Supplementary Material, to the work by Wayne et al.,^40^^,^^41^ or to Ref. 48.

**Table 1:** Technical specifications of the two SPAD cameras used in this study.

Parameter	PF-32	swissSPAD3
Total number of pixels	1024	250,000
Pixel pitch (μm)	50	16.38
Diameter of active area of pixel (μm)	6.9	6
Fill-factor as photo-active area divided by squared pixel pitch (%)	1.5	10.6
Exposure time of single row (ns)	N/A	40
Exposure time of entire array (μs)	3	10

The exposure time of a SPAD array is essential for DCS, as it defines the minimal sampling of the autocorrelation curve (Δτ). The massively parallelized swissSPAD3 is operated by two field programmable gate arrays (FPGAs)—one per sensor half—and the total exposure time $t_{\text{exposure}}$ scales with the number of readout rows in each sensor half at 40 ns per row^40^^,^^41^ for $t_{\text{exposure}}=n_{\text{rows}}\times 40\;\mathrm{ns}$. This configuration essentially results in a fundamental trade-off between the temporal resolution of the $g_{2}$ curves, on the one hand, and the number of independent pixel measurements and thus the increase in SNR, on the other hand. This trade-off is summarized by Wayne et al.^41^ Here, we explored this trade-off in two different experiments.

We employed two different modes of operation to obtain the $g_{2}$ data:

• Streaming of full raw frame data: These data were transferred via an iPASS cable (IPASS PCIE CABLE ASSY 68P 3M, 0745460801, Molex, Wellington, United States) that was connected to a PCIE cable assembly expansion board (AVT-ONIX-PCIE-IPASS-8X-G board, Onix systems, Israel), saving full frame data to a SSD hard drive.
• Automated, real-time, on-board calculation of the $g_{2}$ curves on the respective FPGA unit. The FPGA firmware was developed by Wayne et al.^41^ and outputs $g_{2}(\tau )$ values over a USB3.0 connection at a fixed number of 16 delay times, between $\tau_{0}=0$ (no delay) and $\tau_{\max}$ at 15 times the exposure time.

Both of these configurations enable continuous real-time data streaming. Although the FPGA-based on-board autocorrelation firmware is able to stream the processed $g_{2}$ data continuously over USB3.0, the use of the iPASS cable and the PCIE card enable a continuous stream of full raw data at a data rate of 1.5  Gb/s, which is sufficient to record for hundreds of seconds before encountering memory issues. However, we chose to record relatively short periods (7.5 s of full raw data for the blood flow suppression experiment and 15 s of $g_{2}$ data for the cognitive activation experiment) to reduce the total dataset size of this relatively large scale.

### 2.4 Data Processing

From the detected photon counts, we estimate the normalized intensity autocorrelation, given by

$$g_{2}(\tau )=\frac{\left\langle I(t)\cdot I(t+\tau )\right\rangle }{\langle I(t)\rangle^{2}},$$ (2)

where the brackets ⟨⟩ denote time averages, τ denotes the time delay variable of the temporal autocorrelation, and I(t) is the measured number of binary photon detection events. In each case, this $g_{2}$ was calculated based on Eq. (2) per individual pixel of the sensor.

The temporal resolution Δτ of these $g_{2}$ curves is defined by the total exposure time of each sensor. The 32×32 PF-32 detector has a global shutter, and the exposure time was held constant at 3  μs in all experiments. The larger 500×500 swissSPAD3 detector has a rolling shutter operation where the number of readout rows can be adjusted. Thus, its full-frame exposure time is defined by the number of rows that is read multiplied by the readout time for each row of 40 ns.^40^^,^^41^ In the full raw data streaming mode, the number of rows could be adjusted freely between 1 and 250, for a full-frame exposure time of $t_{\text{exposure}}=n_{\text{rows}}\times 40\;\mathrm{ns}$. The on-board autocorrelation mode only allowed measurements of either 250 rows from one sensor half at 10.81  μs exposure (250×40  ns=10  μs, plus additional wait time for wait states and clock cycle) or 64 rows from two sensor halves (128 rows in total) at 3.37  μs exposure (64×40  ns=2.56  μs, plus additional wait time). For the CBF experiments, where blood flow is generally faster, we used fewer pixels for a quicker exposure time of 3.37  μs. The slower MBF was recorded by more pixels at a total exposure time of 10  μs. In each case, $g_{2}$ was calculated for each individual SPAD pixel and then all curves were averaged across all pixels and across the temporal averaging window ΔT to boost the SNR. An overview of these settings is summarized in the Supplementary Material.

#### 2.4.1 BFI estimation

All calculated $g_{2}$ curves were used to estimate the blood flow index (BFI) according to the semi-infinite solution to the correlation diffusion equation.^49^^–^^54^ We used publicly available code for this model from Wu et al.^55^ Refer to the Supplementary Material for more details. In brief, a closed-form, analytical model for the normalized electric field autocorrelation function, $g_{1}(\tau )$, based on homogeneous tissue properties was used to model the BFI as an effective diffusion coefficient. The respective optical tissue properties were assumed based on literature,^56^^–^^60^ as displayed in the Supplementary Material.

This expression for $g_{1}(\tau )$ was then inserted into the Siegert relation^61^

$$g_{2}(\tau )=1+\beta \cdot |g_{1}(\tau ){|}^{2}.$$ (3)

This analytical expression for the normalized intensity autocorrelation $g_{2}(\tau )$ was fitted to the measured $g_{2}(\tau )$ curve [see Eq. (2)] and optimized for the BFI and β in each individual $g_{2}$ of a given time point ΔT. In the case of the experiment to measure cerebral blood flow at large SDS, only the initial 12 delay values (τ = 3.3 to 40.4  μs) were considered for the BFI estimation, as later delays are usually more noisy (see Fig. 4), as discussed in the Sec. 3. Finally, the residual error of the fitted model was evaluated and a threshold for the median residual of 0.03 was used to exclude outliers from further statistical analysis described below.

Please refer to Table 2 for all details on data recording and BFI reconstruction used for the occlusion experiment on the forearm and for the cognitive experiment on the PFC.

**Table 2:** Technical parameters of data recording (first block) and blood flow reconstruction (second block) in the blood flow suppression experiment in the forearm (left) and in the cognitive activation experiment (right).

	Blood flow suppression experiment in the forearm		Cognitive experiment in the prefrontal cortex	
	Superficial detection (raw data stream)	Deep detection (raw data stream)	Superficial detection (raw data stream)	Deep detection (on-board autocorrelation)
SDS	1.5 cm	4 cm	1.5 cm	4 cm
$N_{\text{pixels}}$	1024	125,000	1024	64,000
Exposure time	3 μs	10 μs	3 μs	3.37 μs
Frame rate	333,333 fps	92,200 fps	333,333 fps	300,000 fps
Temporal avg. window of final BFI (ΔT)	0.1 s	0.1 s	0.125 s	0.125 s
$N_{\text{curves}}$ per ΔT	198	201	744	2295
Delays per $g_{2}$ curve	168	46	56	15
$\tau_{\max}$	504 μs	460 μs	168 μs	49.5 μs

#### 2.4.2 Pulsatility analysis

In this section, we describe the algorithm for measuring pulse frequency as well as pulse markers for the calculation of the pulsatility index and resistance index.

First, a fast Fourier transform (FFT) was carried out of the entire BFI time trace and Matlab’s *findpeaks* function was applied on the FFT data within the upper and lower frequency boundaries of 0.33 to 2.65 Hz or 20 to 160 bpm, a range which is roughly twice as broad as the typical physiological heart rate range for resting adults of about 1 to 1.3 Hz (60 to 80 bpm).^62^ The peak of highest probability (the main peak besides the zero frequency) was identified as the pulse frequency. Then, systolic peaks (SP) were identified as local maximal in the BFI trace, using Matlab’s *findpeaks* function with a minimal distance of 0.6 times the previously determined pulse rate. Diastolic end (DE) points were then obtained as minima in the BFI in between two subsequent systolic peaks. As shown in Fig. 7, the measured pulse rate was generally below 2 Hz, showing that the sampling rates of 8 or 10 Hz in our experiments were always adequate to measure SP and DE. In addition, dicrotic notches (DN) were measured as local minima within a window of 0.4 s following a systolic peak. Diastolic peaks (DP) were identified as local maxima between subsequent DN and DE points. In some cases, the temporal resolution was not sufficient to resolve DPs and DNs so that these two points were identical. These cases were therefore ignored for further analysis. Finally, the pulsatility index (PI) was calculated as^63^

$$\mathrm{PI}=\frac{{\left\langle \mathrm{BFI}\right\rangle }_{s}-\langle \mathrm{BFI}\rangle_{\mathrm{DE}}}{\left\langle \mathrm{BFI}\right\rangle },$$ (4)

where ⟨BFI⟩ is the average BFI of the entire trial and $\langle \mathrm{BFI}\rangle_{\mathrm{SP}}$ and ${\left\langle \mathrm{BFI}\right\rangle }_{\mathrm{DE}}$ denote the respective averages across all detected peaks or end points. Additional pulse metrics are displayed in the Supplementary Material.

### 2.5 Noise Analysis

The abovementioned detection of pulse markers also enables a noise estimation of these *in vivo* data, by considering all peaks (SP) in a BFI trace as subsequent measurements under similar blood flow conditions (respectively all diastolic end points can serve as an additional set of similar blood flow conditions). We then calculated the average noise $\overset{¯}{N}$ of each individual trial as the standard deviation (STD) of each $g_{2}(\tau )$ value, across n data points under similar conditions (i.e., systolic peaks or diastolic end points, respectively)

$$\overset{¯}{N}=\langle N(\tau )\rangle_{\tau }=\langle \mathrm{std}(g_{2}(\tau ){)}_{n}\rangle_{\tau },$$ (5)

where $g_{2}(\tau )$ is the autocorrelation function, which is averaged across all pixels in the detector. The noise was then averaged across all delay values, denoted by the brackets $\langle N(\tau )\rangle_{\tau }$. The distribution of these trial-based noise values was then plotted as boxplot, as seen in Fig. 8. A more detailed noise analysis, shown as a function of τ and as a function of the number of averaged pixels $M_{\text{pixel}}$, can be found in the Supplementary Material and shows the $\sqrt{M_{\text{pixel}}}$ reduction in overall *in vivo* measurement noise.

### 2.6 Statistical Analysis

For each trial, the BFI data were normalized to 100% of the median of the first control measurement. The data from each of the two detectors were analyzed separately so that the short source-detector separation (SDS = 1.5 cm) could serve as an in-built control measurement for the deep PDCS configuration (SDS = 4 cm).

For each individual trial, the median of the time course was calculated for the first control, the test condition (suppression by cuff or activation by cognitive task), and for the second control. The group distribution of these median values in all trials was plotted and analyzed by a linear mixed effects (LME) model to derive p and t values. The LME used the normalized, averaged blood flow metric (relative BFI—rBFI) as the outcome variable while using the trial and the condition (control or experimental) as predictor variables with a random intercept and slope for each trial

rBFI∼Trial+Condition+(Condition|Subject). (6)

We ran the same analysis for both sensors to compare blood flow changes that were recorded simultaneously from superficial and deeper tissue layers. Furthermore, the average difference and Cohen’s difference^64^ of the distribution were calculated. The same procedure was also applied to compare pulsatility parameters between the two detectors. This analysis procedure follows the pre-registered study design.^42^

## 3 Results

In this work, we explored massively parallelized DCS in two different *in vivo* experiments. In the first experiment, we initiated a global decrease of blood flow in the forearm via a pressurized arm cuff and measured PDCS blood flow with our dual-detection system with two different measurement depths. In the second experiment, we placed the same system on the forehead to compare the localized blood flow activity in deeper brain tissue with the more superficial measurement from scalp tissue as a reference.

### 3.1 Sensitivity to Suppression of Muscle Blood Flow in the Forearm

**Fig. 3:**
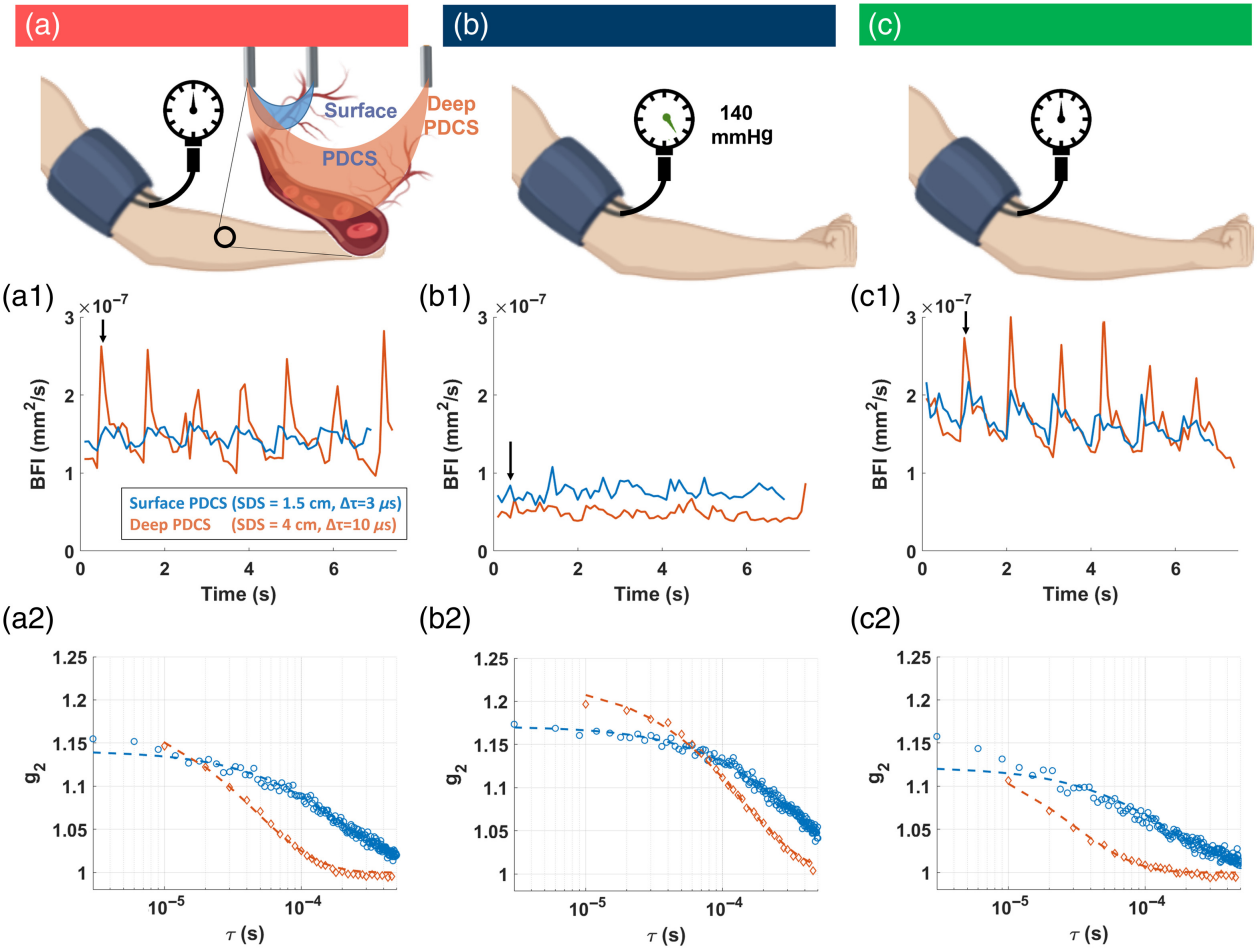
PDCS measurement at the arm of an exemplary subject before (unpressurized, control before) (a), during (pressurized) (b), and after (c) blood flow suppression via a pressurized cuff (unpressurized, control after). Panels (a1), (b1), and (c1) show the blood flow index (BFI). The BFI was sampled at 10 Hz, and each individual value was derived from fitting an analytical model to the averaged autocorrelation curves ($g_{2}(\tau )$). Panels (a2), (b2), and (c2) show one exemplary $g_{2}(\tau )$ that was used to obtained the BFI marked by an arrow in panels (a1), (b1), and (c1). Orange color indicates the deep PDCS measurement (SDS = 4 cm, Δτ=10  μs, averaged across ΔT=0.1  s and 125,000 pixels). Blue color indicates the superficial PDCS measurement (SDS = 1.5 cm, Δτ=3  μs and averaged across ΔT=0.1  s and 1024 pixels).

In the first experiment, we initiated a global decrease of blood flow in the forearm via a pressurized arm cuff. We measured PDCS blood flow with our dual-detection system. For both PDCS configurations (SDS = 1.5 cm and SDS = 4 cm), the derived BFI values [Figs. 3(a1) and 3(c1)] clearly show the characteristic diastolic and systolic peaks that are typically present in arteries,^62^ which confirms blood flow sensitivity for superficial and deep tissue measurements. As expected, the $g_{2}$ curves decay much faster in the PDCS measurements at larger SDS compared with shorter SDS under the same conditions because the longer photon path leads to more scattering events. Upon suppression of blood flow via a pressurized arm cuff [Fig. 3(b)], the $g_{2}$ curves show a later decay and the derived BFI time traces show a significantly decreased BFI overall and no pulsatile behavior.

**Fig. 4:**
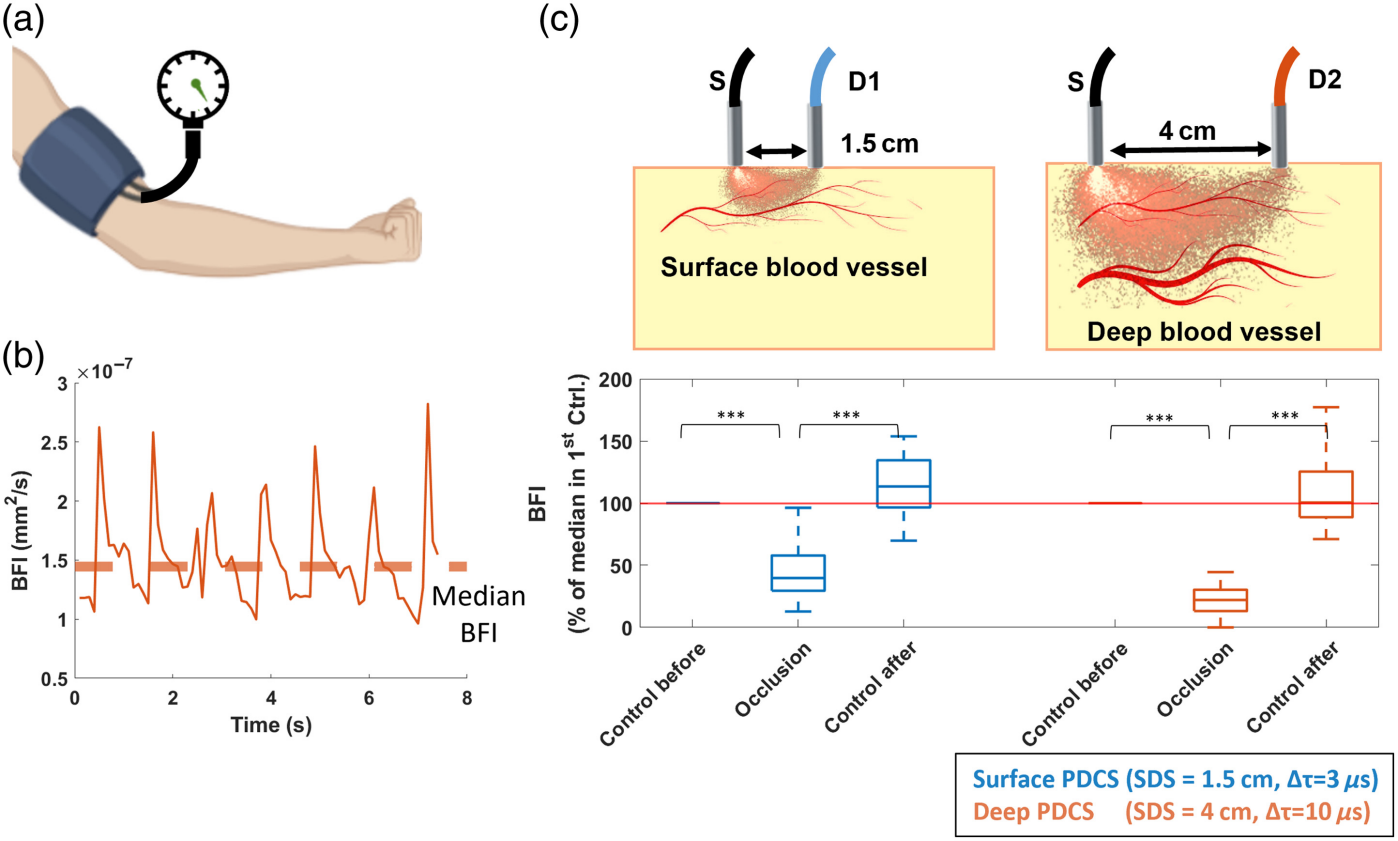
Group data analysis of PDCS measurement (i.e., relative muscle blood flow) during blood flow suppression via pressurized cuff. (a) Flow metrics from suppression experiment. (b) Median BFI. The median BFI value was derived for each individual trial and normalized to the initial control measurement of each trial. (c) Analysis from superficial and deep measurement. The results of all trials were compared between the three conditions (control before, test, control after) and for both detection schemes (superficial PDCS at SDS = 1.5 cm in blue and deep PDCS at SDS = 4cm in orange). This median BFI shows a statistically significant decrease during blood flow suppression (*p<0.05 for all cases). All boxplots show the 25th and 75th percentile as boxes. Significance levels are indicated as *p<0.05, **p<0.01, ***p<0.001. Data include 24 independent trials from 11 subjects.

Figure 4 shows the grouped PDCS results from all trials in the blood flow suppression experiment. These results confirm that the effect observed for an individual trial, described above in Fig. 3, which also holds over a larger group of different subjects. For the superficial PDCS measurement, the suppression of blood flow in the forearm via a pressurized cuff resulted in an average decrease of 50% in normalized BFI [Fig. 4(b)], when compared with the first control. Upon release of the pressure (“Control after”), the normalized BFI reached an average of around 120%, indicating that the normalized BFI exceeded the initial level during reactive hyperemia (i.e., when the blood rushed back). For the deep tissue PDCS measurement, this change is more pronounced, reaching an average decrease of 75% during suppression when compared with the control before or an average difference of 83% when compared with the control after. The results were statistically significant in all conditions (p<0.001).

### 3.2 Sensitivity to Cerebral Blood Flow in the Prefrontal Cortex During Function Task

**Fig. 5:**
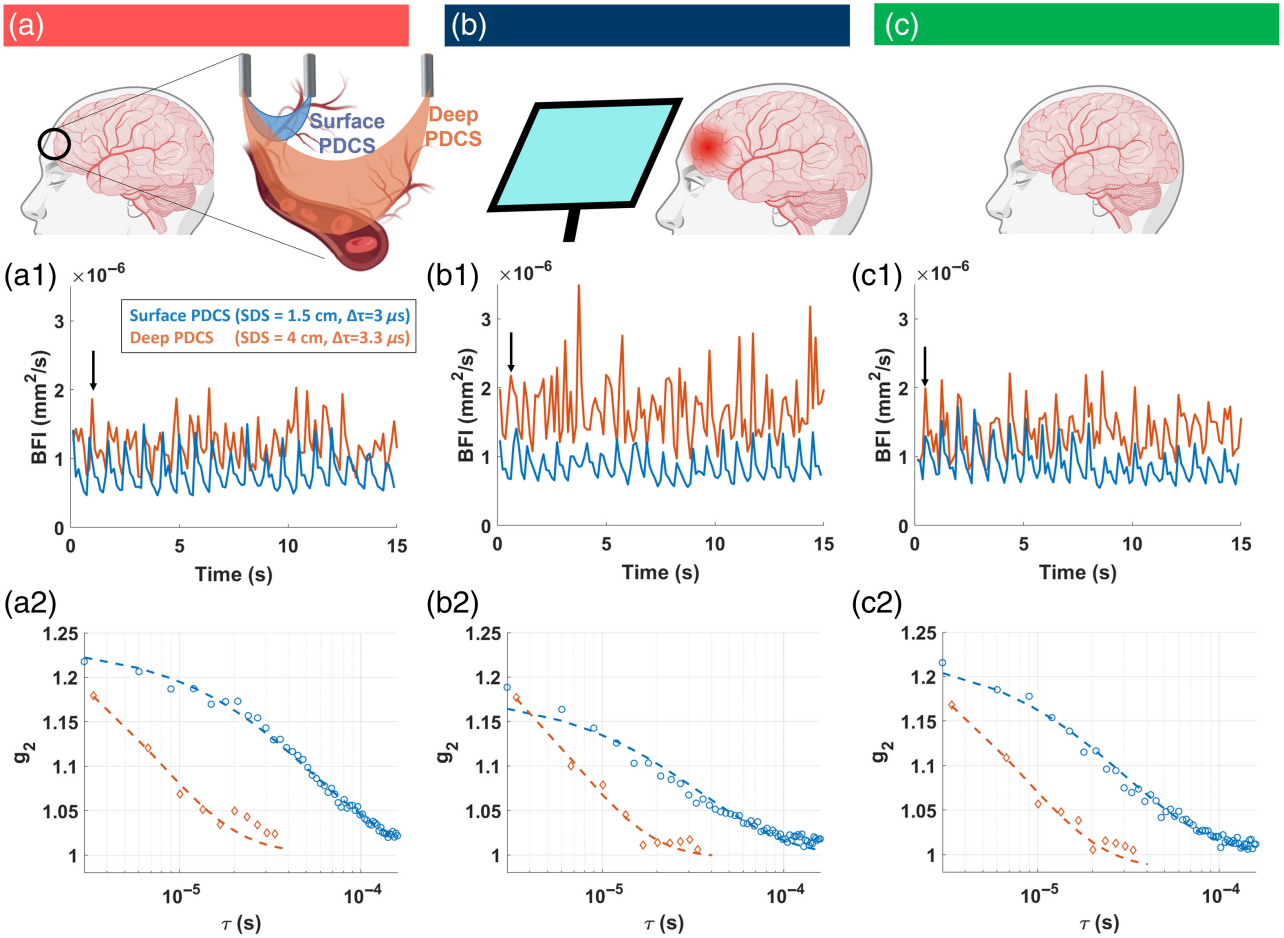
PDCS measurement at the prefrontal cortex of an exemplary subject before (rest state, control before) (a), during (cognitive task, n-back) (b), and after (c) the cognitive task of an n-back test (rest state, control after). Panels (a1), (b1), and (c1) show the blood flow index (BFI) as a function of time. The autocorrelation curves ($g_{2}(\tau )$) were sampled at 8 Hz, and each BFI data point was derived by fitting the averaged $g_{2}(\tau )$ to an analytical model. Panels (a2), (b2), and (c2) show one exemplary $g_{2}(\tau )$ that was used to obtained the BFI marked by an arrow in panels (a1), (b1), and (c1). The orange color indicates the deep PDCS measurement (SDS = 4 cm, Δτ=3.3  μs and averaged across ΔT=0.125  s and 64,000 pixels). The blue color indicates the superficial PDCS measurement (SDS = 1.5 cm, Δτ=3  μs and averaged across ΔT=0.125  s and 1024 pixels).

In the second experiment, we placed the system on the forehead to measure blood flow activity during a cognitive task that is known to induce activity in the prefrontal cortex. Similar to the previously reported results from the forearm, the $g_{2}$ curves from the forehead generally decay much faster at larger SDS [see Figs. 5(a2), 5(b2), and 5(c2)]. The derived BFI values [Figs. 5(a1), 5(b1), and 5(c1)] show pulsatile blood flow at both configurations (SDS = 1.5 cm and SDS = 4 cm), and the peak pulse BFI is higher for the deep PDCS measurement compared with the BFI at superficial PDCS recordings. In contrast to the muscular blood flow in the forearm, the high-SDS configuration used fewer pixels for averaging to decrease the exposure time of the sensor. Thus, the $g_{2}$ curves at larger SDS are noisier as compared with the measurements from the muscle tissue in the forearm, especially toward longer delay values, as seen in Figs. 5(a2), 5(b2), and 5(c2). In this experiment, the derived BFI values [Figs. 5(a1), 5(b1), and 5(c1)] are generally higher for longer source-detector separation, which suggests cerebral sensitivity because it has been reported that BFI can be up to 6 to 10 times higher in the brain as in the surrounding tissue.^65^ During the active cognitive task [Fig. 5(b)], the BFI at large SDS of 4 cm clearly shows an overall increase compared with the control measurement, whereas the BFI that was simultaneously measured at SDS = 1.5 cm shows little to no effect during this task. As shown in Fig. 2(c), this trend is expected if the signal is sensitive to cortical activity and associated blood flow, as a much larger portion of scattered photons will penetrate scalp and skull at an SDS of 4 cm, as compared with an SDS of only 1.5 cm.

**Fig. 6:**
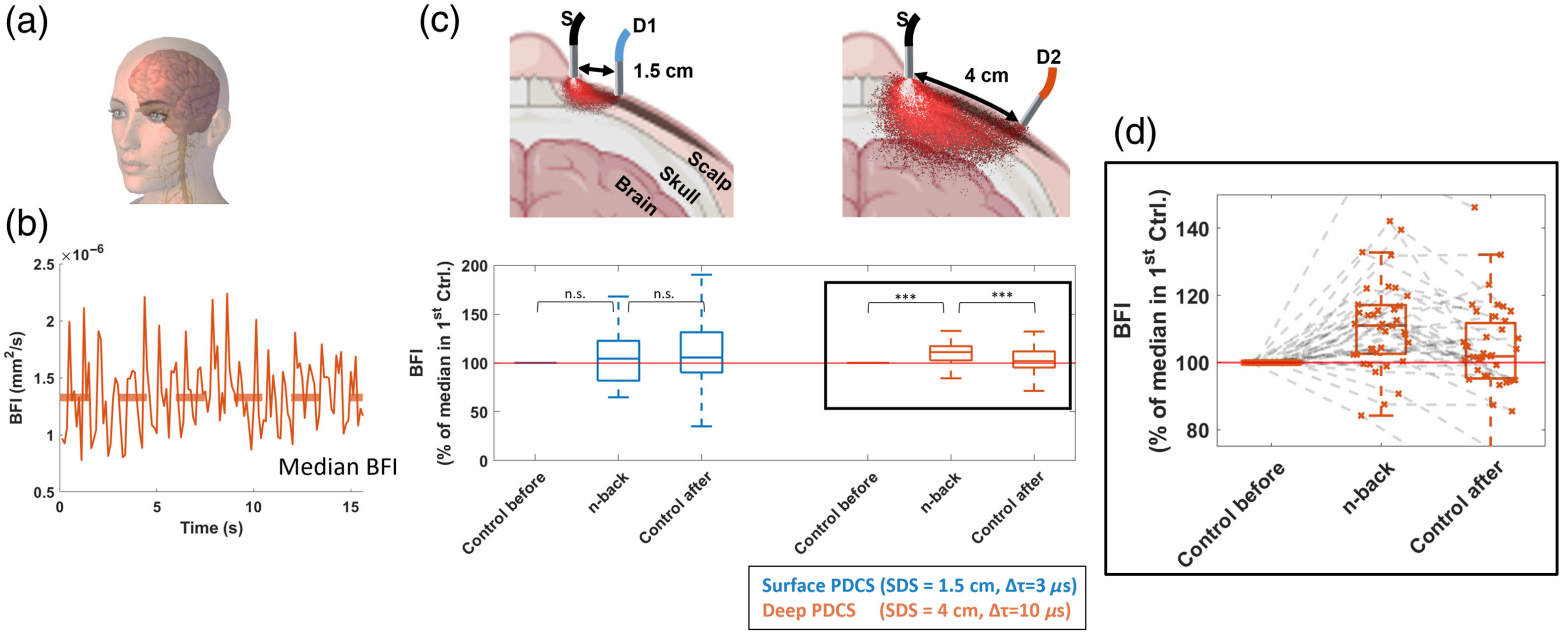
Group data analysis of PDCS measurement (i.e., relative blood flow index) during a cognitive task (flow metrics from cognitive experiment) (a). (b) The median BFI value was derived for each individual trial and normalized to the initial control measurement of each trial (median BFI). (c) The results of all trials were compared between the three conditions (control before, n-back, control after) and for both detection schemes (superficial PDCS at SDS = 1.5 cm in blue and deep PDCS at SDS = 4 cm in orange) (analysis from superficial and deep measurement). Only the deep PDCS configuration shows a statistically significant increase during the cognitive task in both metrics, indicating cerebral sensitivity. Panel (d) shows a re-scaled version of the group differences from the deep tissue layer (at SDS = 4 cm). All boxplots show the 25th and 75th percentile as boxes. The gray-dotted lines in panel (d) show that most trials experienced an increase during the task and again an decrease afterwards, although few individual trials did not yield this result. Significance levels are indicated as *p<0.05, **p<0.01, ***p<0.001. Data include 39 independent trials from 15 subjects.

Figure 6 shows the respective group statistics for the cognitive activation experiment. These results from 15 subjects show the same trend as the BFI in an individual trial, described above in Fig. 5. For the superficial PDCS measurement, the change in normalized BFI did not reach statistical significance in any condition (p>0.05), which indicates little to no change in BFI at the more superficial tissue layers. By contrast, the deeper PDCS measurement at SD = 4 cm reaches statistical significance for the grouped BFI results (p<0.001), with an average increase of 12% and 8%, when compared with the control before or after the cognitive task, respectively [see Figs. 6(b) and 6(c)]. Thus, our experiment provides experimental evidence that our PDCS system at 4 cm SDS can reach cerebral sensitivity in human adults.

In addition to the more rigorous and well-established BFI (Figs. 3 and 5), we tested the initial $g_{2}$ slope as blood flow metric (Figs. S5 and S6 in the Supplementary Material). This metric follows the same general trend in the group statistics as the BFI described above—albeit at a different magnitude.

### 3.3 Pulsatility Analysis

**Fig. 7:**
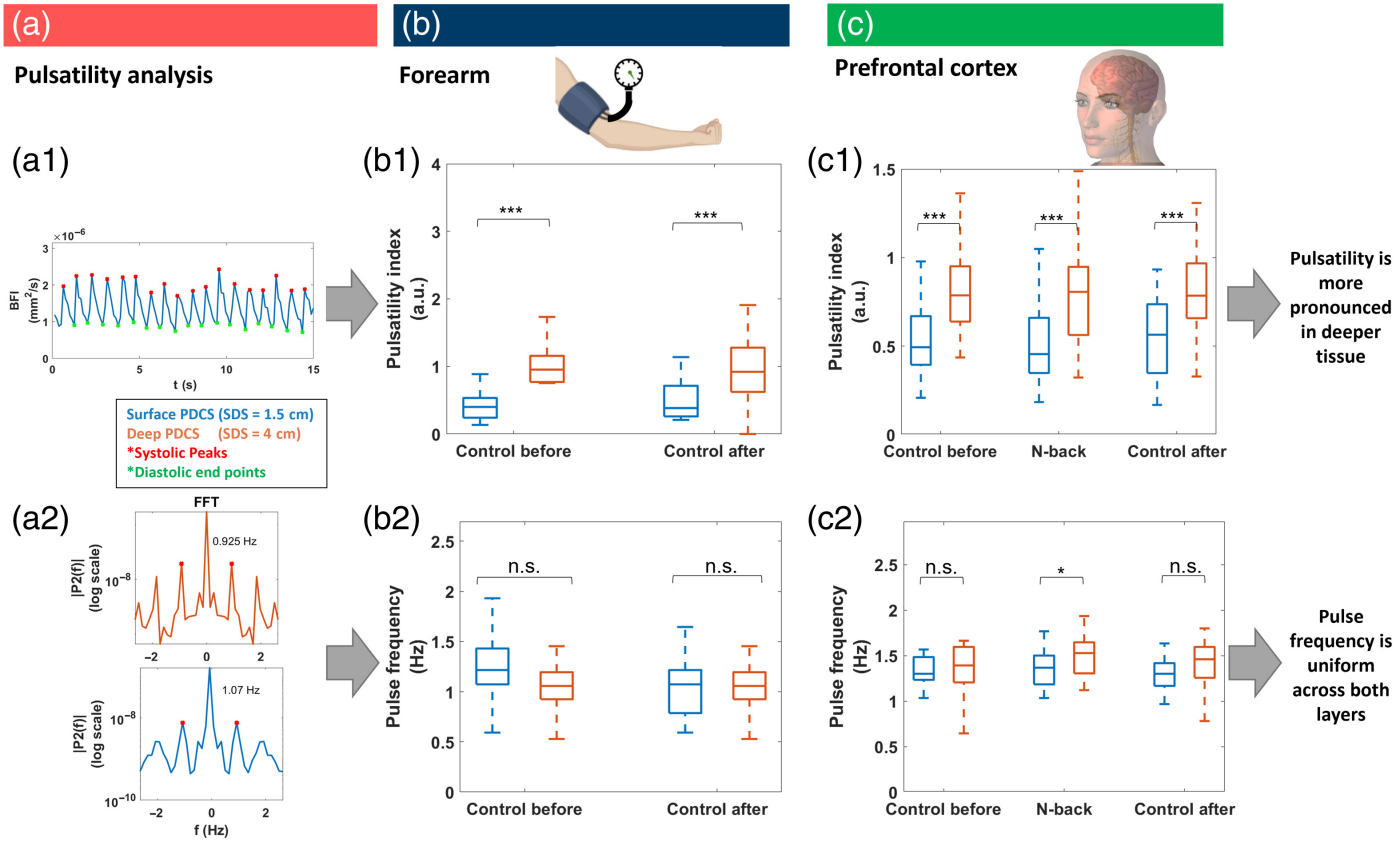
Pulse analysis. (a) Our algorithm detects systolic peaks, diastolic endpoints, dicrotic notches, and diastolic peaks in data from both SDS (a1), as well as the pulse rate (a2, both examples recorded at 10 Hz from the same experiment). Based on these metrics, the pulsatility index (b1, c1) was calculated and analyzed statistically across all measurements for the suppression experiment in the forearm (b), as well as for the cognitive experiment (prefrontal cortex) (c). Data during suppression of blood flow in the arm are not shown because there was no pulsatile flow in this case. Significance levels are indicated as *p<0.05, **p<0.01, ***p<0.001. Data include 33 independent trials from 11 subjects in panel (b) and 39 trails from 15 subjects in panel (c).

A qualitative inspection of all BFI time traces [representative examples shown in Figs. 3, 5, and 7(a1)] suggests that the BFI from the superficial PDCS measurement shows lower BFI peak values than the deep PDCS measurement. To quantitatively test this observation, we developed software to detect pulse markers of systolic peaks, diastolic endpoints, dicrotic notches, and diastolic peaks [Fig. 7(a1)] and the pulse rate [Fig. 7(a2)] in the pulsatile BFI traces. Based on these pulse markers, we calculated the pulsatility index (PI), as described in the methods section above (additional metrics of resistance index and notch index are shown in the Supplementary Material). Figures 7(b) and 7(c) show the statistical analysis of these parameters across all trials, comparing the superficial measurement (SDS = 1.5 cm, blue) to the deeper measurement (SDS = 4 cm, orange).

In the case of the global flow suppression in the arm as shown in Fig. 7(b), the PI [Fig. 7(b1)] showed clearly significant increases at greater SDS as compared with the reference at lower SDS. The PI was increased by an average of 0.44 to 0.49 in both control measurements (Cohen’s D=1.1 to 1.2, $p<10^{-4}$). The pulse rate however [Fig. 7(b2)] showed no notable differences within the measurement accuracy. All data of the 25th to 75th percentile from the two simultaneous detection schemes were within a range of 0.8 to 1.4 Hz or 48 to 84 bpm (Cohen’s D=−0.3 to 0.1, p>0.2), which relates well to the expected physiological range for resting heart rate in healthy adults of about 1 to 1.3 Hz (60 to 80 bpm).^62^ The test condition during blood flow suppression was excluded because there was minimal pulsatile flow during suppression.

Please note the difference of ∼0.15  Hz [Fig. 7(a2)] in simultaneously measured pulse rates from both detectors during the flow suppression experiment (7 s intervals) could be caused by the offset of only a single pulse between both detectors, which could occur because both detectors were not perfectly synchronized (eight pulses within 7.5 s result in 1.07 Hz while seven pulses in 7.5 s result in 0.925 Hz). This level of uncertainty can explain some of the variability of the pulse rates in Figs. 7(b2) and 7(c2). Accordingly, the offset of a single pulse in the cognitive experiment (15 s intervals) would result in an uncertainty of ∼0.06  Hz.

The results for PI in the PFC experiment [Fig. 7(c1)] generally follow a similar trend of significantly increased pulsatility at greater SDS. The PI was increased by 0.28 (Cohen’s D of 1.1 to 1.2, $p<10^{-4}$) for all three conditions. The measured pulse rate [Fig. 7(c3)] showed that all data within 25th to 75th percentile were within a range of 1.1 to 1.4 Hz or 66 to 84 bpm (Cohen’s D<0.5, p>0.01) and showed no significant difference within the measurement accuracy for the two control cases. During the cognitive task, our data slightly reached statistical significance with an average increase of 0.15 Hz or 9 bpm higher between lower SDS and larger SDS (Cohens D=0.53, p<0.03). We assume that the uncertainty from the offset of a single pulse, as discussed above could explain some of this unexpected difference.

### 3.4 Noise Analysis

**Fig. 8:**
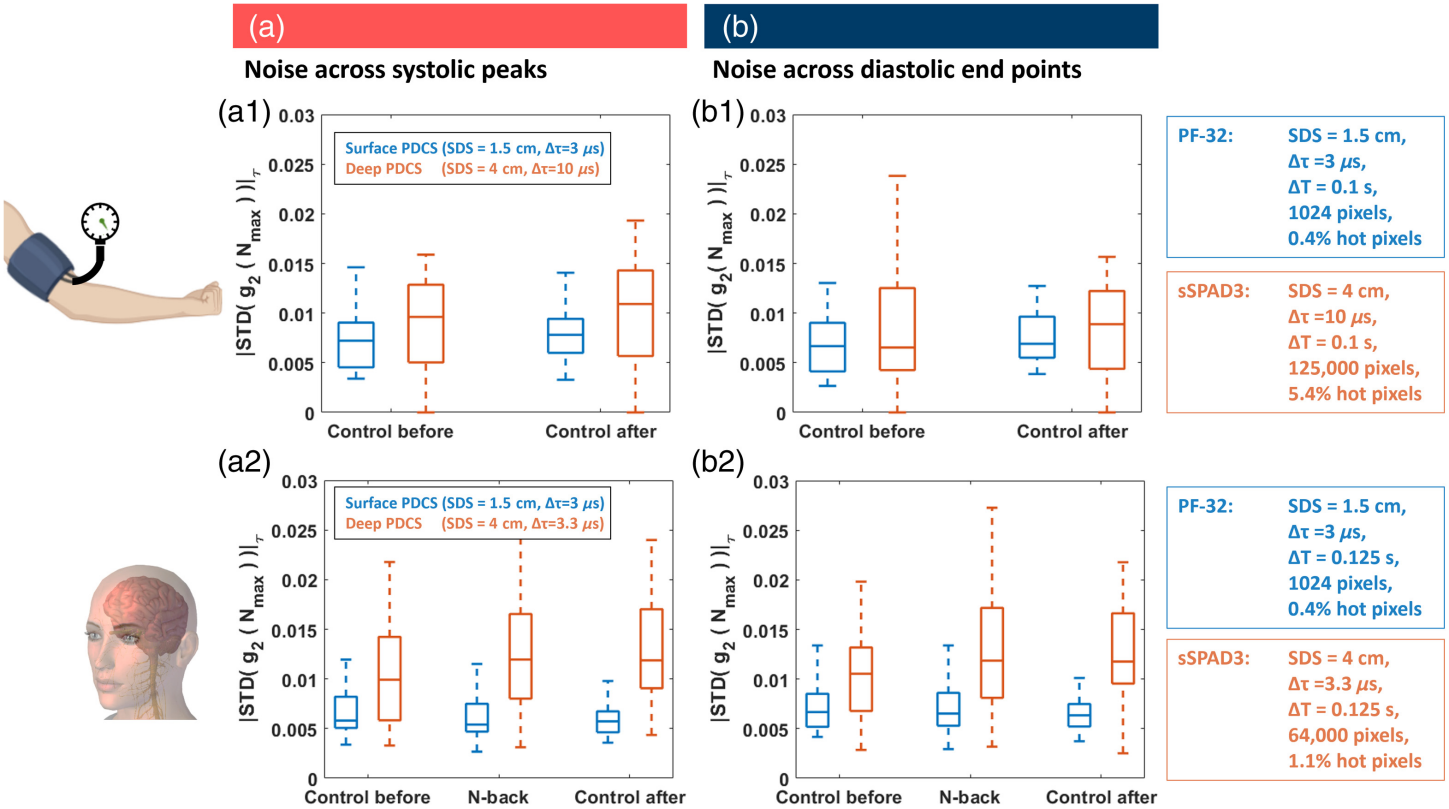
New 500×500 array can enhance the measurement depths in PDCS by achieving a similar noise level at 4 cm SDS as the previous PDCS with a 32×32 array at only 1.5 cm SDS. Experimental noise was calculated as the standard deviation (STD) of the $g_{2}$ curves across all subsequent systolic peaks (a) or diastolic endpoints (b). Results from the global blood flow suppression in the forearm (a1) and (b1) show that the noise from the 32×32 array at 1.5 cm SDS and 3  μs exposure is on the same level as that of the larger detector with 125,000 pixels at 4 cm SDS and 10  μs exposure. In the experiment on local activation in the PFC (a2) and (b2), the noise reduction of the larger detector was deliberately sacrificed by recorded only 64,000 pixels to achieve a shorter exposure time of 3.3  μs and thus a better flow sensitivity at the cost of increased noise (smaller Δτ). Data include 33 independent trials from 11 subjects for forearm and 39 trails from 15 subjects for the cognitive experiment.

The detection of pulse markers shown above was also leveraged to facilitate a noise analysis in our *in vivo* PDCS measurements. The noise in DCS can be calculated as the standard deviation (STD) of the $g_{2}$ curves across subsequent measurements under the same experimental conditions. In past experiments on SPAD arrays, this assessment has been carried out in optical phantom experiments under “static” conditions (i.e., only considering a diffusion coefficient in the medium).^37^^,^^41^^,^^66^ Based on the detection of systolic peaks and diastolic endpoints [Fig. 7(a)], we calculated the noise in our data as the STD across subsequent systolic peaks [maximal flow, Fig. 8(a)] or diastolic endpoints [minimal flow, Fig. 8(b)] in each trial. This noise metric can be interpreted as the holistic combination of all possible sources of noise, including technical *and* biological variations in the measurements, across subsequent blood flow peaks. In Fig. 8, the distribution of this metric is then plotted across all trials.

Results from the global blood flow suppression in the forearm [Figs. 8(a1) and 8(b1)] show that the noise of the detector with 1024 pixels at 1.5 cm SDS and 3  μs exposure is on the same level as that of the larger detector with 125,000 pixels at 4 cm SDS and 10  μs exposure. In the experiment on local activation in the PFC [Figs. 8(a2) and 8(b2)], the noise reduction of the larger detector was deliberately sacrificed by recording fewer pixels to achieve a shorter exposure time and thus a better sampling of the autocorrelation curve decay (smaller Δτ). In this case, the median noise of the larger detector at 4 cm SDS is generally about 2 or 3 times as high as that of the smaller detector at 1.5 cm SDS. Please refer to the Supplementary Material for more details, as well as a pixel-level noise analysis.

## 4 Discussions

This study utilized multiple large pixel-count SPAD array cameras to perform parallelized diffuse correlation spectroscopy (PDCS) with *in vivo* experiments on human subject with a robust sample size. Although previous the state-of-the art in *in vivo* PDCS applications used much smaller array with only 1024 parallelized detections, we showcase the use of up to 125,000 parallelized detection channels for a much more substantial boost in SNR. This approach allowed us to record deep-tissue, pulsatile blood flow in the head and muscle with an SDS of 4 cm (measured from the center of the illumination spot to the center of the detection fiber) and a sampling rates of 8 to 10 Hz, which is on par with the current front-runners in DCS technology.^22^^,^^35^ By applying it in a relatively large study, we were able to confirm functional CBF changes during a memory task that follow expected trends and to estimate the real, *in vivo* noise level of such measurements.

In contrast to most previous studies on new DCS prototypes, we present a study cohort of more than a dozen adult subjects under real experimental conditions, which allowed robust statistical analysis of all of our findings. In summary, our system is sensitive to global suppression of blood flow, as well as to a localized blood activation in the brain, which was validated in part with the use of a second simultaneously acquired PDCS reference measurement at lower SDS. Furthermore, we were able to provide strong experimental evidence for an increase pulsatility and resistance in deeper PDCS measurements (greater SDS), and we could quantify the overall noise in these measurements, including technical and biological variations.

### 4.1 Trade-off Between SNR and Temporal Sampling of Autocorrelation Curves

The fidelity of massively parallelized DCS is governed by the trade-off between temporal resolution of the autocorrelation function $g_{2}(\tau )$ and the signal-to-noise ratio (SNR) in photon detection. In PDCS, the exposure time of each SPAD pixel defines the temporal sampling of these $g_{2}$ curves (Δτ). A fast exposure time is essential for deep-tissue measurements where the average number of scattering events increases towards many thousands, which causes the temporal speckle fluctuations to increase to the MHz regime. The shutter of the SwissSPAD3 array defines the total exposure time by the number of rows that are read out. More rows allow more parallelized measurements and thus higher SNR, but result in longer exposure times, which increases Δτ and thereby inhibits sensitivity to faster and deeper flow.

Our results from the arm showed that it is possible to acquire PDCS measurements with 125,000 pixels (250 rows from one sensor half for 250×500  pixels) from the new swissSPAD3 array at 4 cm SDS. A similar blood flow occlusion experiment was recently shown by Della et al.^67^ Our in-depth analysis show that this massivley parallelized SPAD array at 4 cm SDS offers a similar noise level as the conventional 1024 pixel array at 1.5 cm SDS (note: the diameter of the active sensor area is similar for both arrays with 6.9  μm for PF-32 and 6  μm for swissSPAD3, temporal averaging window was identical at 0.1 s in both detection schemes). However, this came at the cost of reduced temporal resolution of the $g_{2}$ curves for the larger array of 10  μs compared with the 3  μs of the smaller PF-32. Nevertheless, both detectors still adequately sampled the autocorrelation decay the resulting BFI drop during flow suppression in the forearm.

The issue of reduced sensitivity to deeper and faster flow caused by longer integration time Δτ, and thus a more sparse sampling of the autocorrelation, becomes much more critical for the measurement of CBF in the PFC, where the $g_{2}$ decays faster than in the muscle. Therefore, we employed a new onboard autocorrelation calculation of only 64,000 pixels (64 rows from each of the two sensor halves for 128×500  pixels) to reduce Δτ. To slightly compensate for the respective reduction in SNR, we used a larger temporal averaging window ΔT of 0.128 s and fewer τ data points per $g_{2}$ curve to average a larger number of subsequent autocorrelation curves. Although this procedure reduced the temporal sampling rate of the BFI trace, it still allowed detection and analysis of pulsatile blood flow while maintaining similar exposure times for both arrays (Δτ=3.37  μs at 4 cm SDS versus 3  μs at 1.5 cm SDS).

These settings allowed us to clearly measure pulsatile blood flow in both experiments and to perform a more granular analysis of pulsatile metrics (Fig. 7), as compared with the mere median blood flow metrics shown in Figs. 4 and 6.

### 4.2 Pulsatility Analysis

According to our experimental data in Fig. 7, the measured pulsatility index seems to be generally higher for greater measurement depth of our PDCS system, as compared with the more superficial measurement at shorter SDS. We do not believe that this observation is an effect of different data qualities in the different configurations because all available data quality metrics (BFI model residual error in Fig. S3 in the Supplementary Material or the overall $g_{2}$ noise in Fig. 8 and in Figs. S8 and S9 in the Supplementary Material) indicate a similar measurement noise and fit accuracy. One potential, but still entirely speculative interpretation, could be that superficial measurements include a higher ratio of smaller capillaries, whereas the deeper PDCS measurement at SDS = 4 cm can reach more of the larger arteries in deeper tissue layers. Blood flow velocity and pulsatility differ greatly and nonlinearly across different vessels, such as arteries, arterioles, capillaries, and veins, with different vessel diameter, wall thickness, and overall flow resistance.^62^ However, the exact relation between these factors and the measured BFI, as the shear-induced diffusion coefficient of red blood cells, in (P) DCS measurements is still unclear and would be interesting for future research.

Although this work demonstrated 8 to 10 Hz pulse sampling rates, we believe that the SNR-gain in PDCS can enable faster sampling rates to resolve even higher frequency BFI features, e.g., at 20 Hz^68^ or even at 100 Hz^69^ in the future.

#### 4.2.1 Dual detection in PDCS

The utilization of two different detectors at distinct SDS facilitated the comparison of blood flow changes at shorter SDS (superficial tissue layers) with those at greater SDS that also reach deeper tissue. Thereby, the detector at short SDS served as an in-built comparison measurement under the exact same experimental conditions. As expected, both detectors show a clear drop in blood flow during global suppression in the forearm. However, only the deep PDCS configuration at greater SDS showed significant increases in blood flow during the cognitive task, whereas the reference measurement at lower SDS showed no significant change. This result is expected if cerebral sensitivity was reached at the large SDS, whereas the smaller SDS is mostly sensitive to extracerebral scattering in scalp and skull. The dual detection scheme allowed to rule out other potential causes for increased blood flow at the large SDS not assigned to cerebral activity, such as motion artifacts, or changes in blood pressure and heart rate, while also allowing a noise estimation for both configurations. Although similar approaches of using multiple detection systems for different measurement depths have been already been used in DCS,^4^^,^^5^^,^^20^^,^^70^ its application in PDCS with entire arrays of SPADs is new.

Attempts to subtract the extracerebral contributions from the scalp and skull in deep measurements have already been translated from fNIRS to DCS,^54^^,^^70^ with a great body of work on multi-layer models (MLM) for DCS.^71^ However, it has also been stated that homogeneous models perform better under general assumptions and that MLM “should be taken with caution.”^71^ The use of more complex MLMs with a greater number of model parameters can be especially challenging for PDCS with large SPAD arrays, in which the minimal delay value Δτ, and therefore the number of delay values until complete decorrelation ($g_{2}=1$), is limited by the exposure time of the array. Compared with simpler semi-infinite models with homogeneous optical properties, MLMs are more prone to result in ill-posed problems because they include a greater number of parameters (e.g., distinct optical properties in each slap). In our experimental data (e.g., 46 or 168 delay values in the arm experiment and 15 or 56 in the PFC, respectively, see in the Supplementary Material), these MLMs did not yield reliable results for multilayered BFI estimation.

In the future, faster SPAD array technology, in combination with more sophisticated data processing techniques, might be able to leverage such dual-detection PDCS data to minimize the contributions of the extracerebral layers to the large-SDS measurement to isolate the component of the signal that arises purely from the cerebral tissue at greater depth. We believe that these techniques could include additional information about the vasculature at the respective tissue depth, as well as mathematical models on how this vasculature shapes the flow dynamics.

#### 4.2.2 Limitations and challenges

The study acknowledges potential limitations and challenges in terms of the underlying biology, the technical setup, and data processing. With respect to the technical setup, we recognize the limitations in SNR-versus-flow sensitivity trade-off that were already discussed above. Another technical limitation is the PDE of both SPADs arrays, which is ∼4 to 5 times lower than in some of the best single SPADs (e.g., PDE ∼15% at 785 nm in arrays against up to 70% in single SPADs).^16^ However, the extremely large number of independent pixel measurements more than compensates this effect. In the future, this effect could even be reduced by using a micro lens array, which was not used in this study, but is generally available for this device.

In addition, there is a natural variance in all biological parameters, such as scalp and skull thickness, pulse rate, or pulse-to-pulse variations. For instance, given the known variation in extracerebral layer thickness across individuals, our described methodology might yield varying cerebral sensitivity for each individual subject. Indeed, the median BFI did not indicate cerebral sensitivity for every individual trial, although the general group difference did show a notable effect [see zoomed inlet of Fig. 6(b)].

It must be noted that mismatches between the assumed optical properties, that relied on reasonable estimations commonly used in literature (see Supplementary Material), and the actual properties of the respective subject would translate to errors in the estimated BFI values. The absorption parameter $\mu_{a}$, in particular, has been described to vary largely among different skin color types (i.e., a 5-× difference in melanin concentration),^72^ and separate values for each individual subject should be taken into account for future experiments.

Another potential limitation arises from using short integration windows for the autocorrelation calculation, which can result in biased results for $g_{2}(\tau )$. Although this issue is fundamental to any $g_{2}(\tau )$ estimation from a finite series of intensity data, it is rarely relevant in conventional DCS, where the integration window is typically increased to increase SNR. Because our massively parallelized DCS technique instead boosts the SNR by averaging individual pixels of a SPAD array, it enables short integration windows at high SNR, which could induce a bias to the $g_{2}(\tau )$ estimation. At an autocorrelation integration window of 0.0615 s (in case of the PFC experiment), we occasionally observed biased $g_{2}(\tau )$ data. To ensure convergence of the conventional BFI model, we corrected this offset before fitting (see Supplementary Material). Although this bias correction might affect the accuracy of the absolute BFI value, our statistical analysis of effect size only considered the relative changes in normalized BFI values, which should be unaffected by this procedure.

Finally, some measurements were discarded based on a pre-defined exclusion criterion according to the residual error of the BFI model fit to the data. In total, 24 participants completed a total of 47 trials of the n-back test and 33 trials of the forearm MBF occlusion. Of these, 39 PFC trials passed the BFI fitting criterion (83%) and 24 trials passed it in the forearm experiment (73%). In total, only about 20% of trials (17/80) were discarded.

In summary, we acknowledge some potential shortcomings that especially may have impacted the accuracy of the absolute BFI values and thus limit our conclusions to relative BFI changes. We aim to adopt a more precise system calibration procedure to help address such limitations in future work.

## 5 Conclusion

Already in 2020, a one Mpixel SPAD camera was reported, albeit at a relatively low frame rate of 24 kfps.^73^ Our study used the latest swissSPAD3 camera with 0.25 Mpixel at 97 kfps (or fewer pixels at higher rate), which allowed continuous transmission of the full raw data due to the addition of an iPASS cable.^40^ Devices of similar pixel size and pixel count are already commercially available, and this ongoing development of SPAD camera technology will likely result in larger, faster, and more affordable detectors in the future. This would allow to sample the $g_{2}$ curves at higher temporal resolution (smaller Δτ) for higher blood flow sensitivity while also allowing more parallelized DCS measurements to increase the SNR even further. These advances would allow higher sampling rates (smaller averaging windows) of the blood flow metrics, higher temporal resolution of blood flow dynamics, and ultimately even deeper DCS measurements. In addition, new data processing techniques, tailored for the sparse signal of binary photon detection events in SPAD detectors are an anticipated refinement for the future. This study provides experimental evidence in using massively parallelized SPAD cameras to measure cerebral blood flow *in vivo* and therefore lays the groundwork towards advancing physiological research and clinical diagnostics in the future.

## Supplementary Material

10.1117/1.NPh.12.2.025007.s01

## Data Availability

The code for the diffusion model was based on the scatterBrains project from Wu et al.,^55^ which is publicly available at https://github.com/wumelissa/scatterBrains. Due to the size of the raw data, the data are only available from the corresponding author upon reasonable request.
