# Supplementary material for: Beneath the surface: revealing deep-tissue blood flow in human subjects with massively parallelized diffuse correlation spectroscopy
Source: Neurophotonics. 2025 Apr 9;12(2):025007. doi: 10.1117/1.NPh.12.2.025007 (PMC11981687; doi:10.1117/1.NPh.12.2.025007)
Supplement: Supplementary file 1 [file NPh_012_025007_SD001.pdf]

Supplementary to: Beneath the Surface: Revealing Deep-Tissue Blood Flow in Human Subjects with Massively Parallelized Diffuse Correlation Spectroscopy

Lucas Kreiss[1*], Melissa Wu[1], Michael Wayne[2], Shiqi Xu[1], Paul McKee[3], Derrick Dwamena[3], Kanghyun Kim[1], Kyung Chul Lee[1,4], Kyle Cowdrick[5], Wenhui Liu[6], Arin Ülkü[2], Mark Harfouche[7], Xi Yang[1], Clare Cook[1], Seung Ah Lee[4], Erin Buckley[5], Claudio Bruschini[2], Edoardo Charbon[2], Scott Huettel[3], Roarke Horstmeyer[1,7]

[1]Department of Biomedical Engineering, Duke University, Durham, NC 27708, USA
[2]Advanced Quantum Architecture Laboratory, École polytechnique fédérale de Lausanne (EPFL), Neuchatel, NE 2000, Switzerland
[3]Department of Psychology and Neuroscience, Duke University, Durham, NC, USA, 27708
[4]School of Electrical & Electronic Engineering, Yonsei University, Seoul, 03722, Republic of Korea
[5]Georgia Institute of Technology and Emory University, Wallace H. Coulter Department of Biomedical Engineering, Atlanta, Georgia, USA
[6]Department of Automation, Tsinghua University, Beijing, China
[7]Ramona Optics, Inc., Durham, NC 27708, USA

*Lucas Kreiss, lucas.kreiss@duke.edu

1 Demographics of study participants

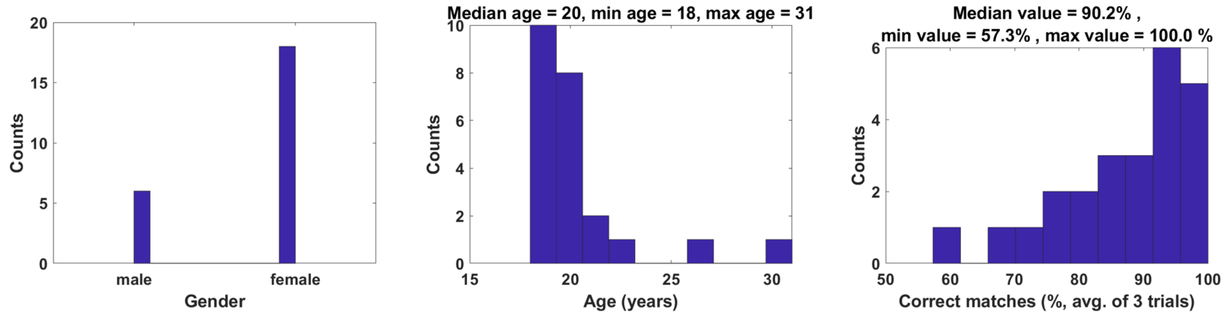

Fig S1 Demographics (Gender and Age), as well as average test performance of subjects in this study.

Study participants were selected from a pool of volunteers with no affiliation to the laboratory. Each subject was briefed about the study protocol and filled out a consent form before participation. Additionally, subjects provided information about their main demographics of sex, age and ethnicity, as summarized in Figure S1 and Table 1. Furthermore, the results of the n-back task (n=2) were documented. This task show a series of 25 letters, and usually contained around six instances, where the same letter was repeated from two letter before. If subjects correctly indicated

Subject ID	Sex	Age	Ethnicity	n-back score
90046	M	19	Multiple	78.86
89815	M	19	Multiple	100
89632	NA	NA	Native American	91.66
89374	F	18	South Asian	67
89869	F	20	East Asian	60.6
89791	F	20	African	95.3
89596	F	21	Caucasian	94.3
89023	F	20	Native American	100
90160	F	20	East Asian	74.3
90166	F	19	East Asian	77.6
89941	M	19	Caucasian	82.3
89548	F	20	East Asian	89.6
87322	M	18	East Asian	94.3
00001	F	31	Middle East	96.3
89998	F	21	Caucasian	100
90055	F	20	Hispanic	86.6
89368	M	19	Hispanic	90.6
89701	F	20	Caucasian	95.3
87337	F	18	African	88.6
89908	F	19	Hispanic	85
89875	F	18	Caucasian	100
00002	M	26	South Asian	83
89539	F	18	Multiple	94.3
00003	F	23	Hispanic	73.6

Table 1 Main demographics of all volunteering subject in this study. NA = Statement not available, no statement made. N-back score stated as % of correct matches, mean over 3 trials

this instance, it was counted as correct match. The percentage of correct matches, the percentage of missed matches and the percentage of false alarms were tracked. Figure S1 and Table 1 show the average percentage of correct matches over all three trials per subject. The recruited subjects come from very diverse ethnicities and have a median age of 20. 70 % of all participants were female (17/24) and 25 % were male (6/24). The median test score of the task was 90.1 %.

Deep pDCS data processing during blood flow suppression in the forearm In the blood occlusion experiment at the forearm, deep tissue recordings (at SDS = 4 cm) were obtained by one full half of the swissSPAD3 detector (250 x 500 pixels) in full raw data stream mode, resulting in

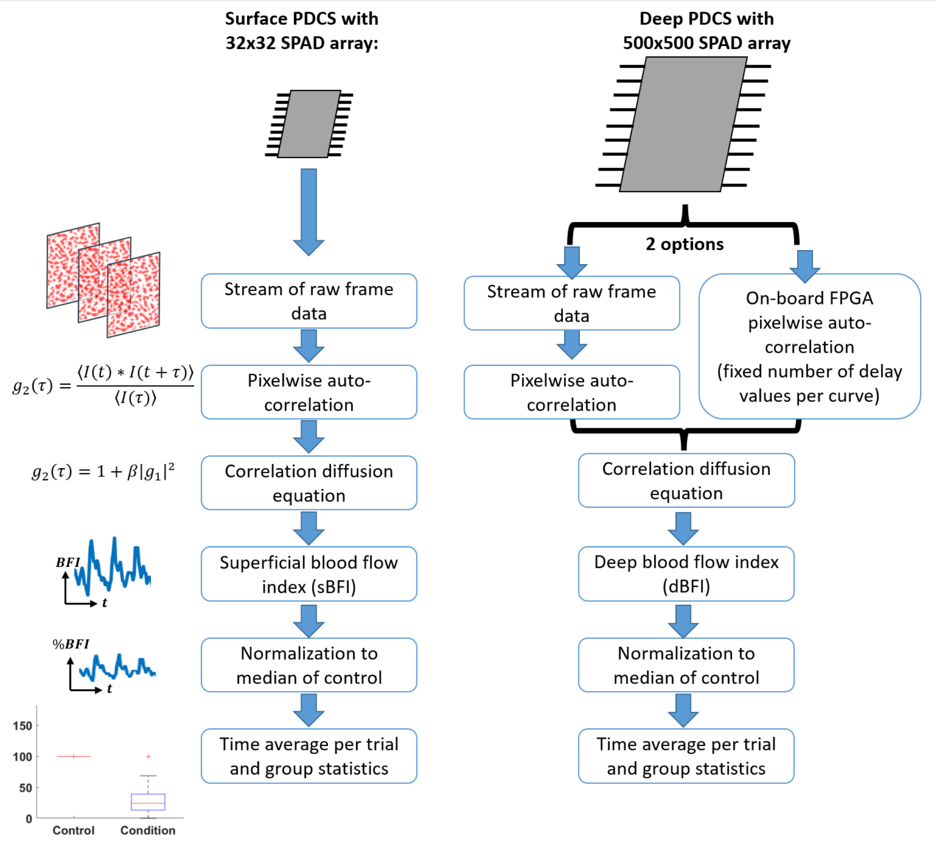

Fig S2 General overview of the data processing pipeline. The PDCS data from the 32x32 SPAD array was always analyzed with the same procedure. The PDCS data from the 500x500 SPAD array was used in two different modes of operation. For further details see the main text.

an exposure time of $250 \times 40ns = 10\ \mu s$. Here, the full raw data was streamed via iPASS cable, as described above. The g_2 calculation was then performed in post-processing at 46 different delay times ($\tau_{max} = 46 \times 10\ \mu s = 460\ \mu s$). Respectively, the g_2 calculation for the superficial pDCS measurements, using the PF-32 detector at $SDS = 1.5\ cm$ and $3\ \mu s$ exposure was carried out at 168 different delay times to get a similar range of delay times ($\tau_{max} = 168 \times 3\ \mu s = 504\ \mu s$). In the case of the occlusion experiment, all autocorrelation curves from both detectors were averaged within an averaging window of 0.1 s (sampling rate of 10 Hz).

Deep pDCS data processing during blood flow activation in the prefrontal cortex In the case of the measurement at the PFC, the optical path in the deep measurement configuration (i.e., long

source-detector separation) crosses the skull and reaches the brain tissue (see Fig. 2 c in the main text). Similar to the previous case, the data at larger SDS shows faster decorrelation compared to the lower SDS data, as expected. Compared to the muscle tissue in the forearm however, the tissue at the forehead has a higher probability for scattering, which increases decorrelation at large SDS even further. This is also supported by our evidence shown in Figures 3 a & 4 a of the main manuscript.

As explained above, reading out all 250 rows would result in an exposure time of $10\ \mu s$, which is relatively long for the faster decay in brain tissue. Thus, there is a trade-off between the increase in SNR (more rows) on the one hand, and the resolution of the g_2 curves ($\Delta\tau$), which relates to the blood flow sensitivity in brain tissue, on the other hand. We addressed this trade-off by using a new, customized on-board FPGA autocorrelation calculation of a quarter of the rows of the full array (64 rows per sensor half, 128 rows in total), developed by Wayne et al. (see Ref. [40] in main manuscript), at an exposure time of $3.37\ \mu s$ ($64 \times 40ns = 2.56\ \mu s$, plus additional wait time). This firmware uses a hard-coded 4×255 exposures for each single g_2 curve with 16 delays, which defines the minimal integration window per g_2 curve at $\Delta t = 4 \times 3.37\ \mu s \times 255 = 3.4ms$. The data in this experiment regarding cognitive activity in the PFC was recorded in 18 times this minimal integration window, resulting in an averaging window of 0.0615s or in a total of 18,360 pixel-wise recordings for each g_2 curve with 16 delay values. In post-processing, these $g_2(\tau)$ data were again averaged 20-fold for a final window of 0.1237s per BFI value or a BFI sampling rate of $\frac{1}{0.123s} \approx 8\ Hz$.

Since this on-board autocorrelation had a fixed number of 16 delay values (including $\tau_0 = 0$) the maximal delay time was defined at $\tau_{max} = 15 \times 3.37\ \mu s = 50.55\ \mu s$. Occasionally, we observed a bias in the autocorrelation, leading to the g_2 curve decaying to values other than 1.

In those cases, we corrected this offset by adding the final value $|g_2(\tau_{max}) - 1|$ to all g_2 values, before fitting by the BFI model. Our group is currently working on future research to determine a reliable quantification of the minimally required integration window and number of associated photon detection events to truly warrant an unbiased autocorrelation calculation.

As seen in Fig. 5 of the main text, the g_2 data in the cognitive experiment showed larger variations at late delay values. In order to reduce the effect of this noise on the BFI estimation and to emphasize the earlier delay values of higher blood flow sensitivity, the analytical g_2 model (see next section below) only used the first 12 delay values of the autocorrelation, ranging from $3.37\ \mu s$ to $40.44\ \mu s$.

The corresponding superficial measurements with the PF-32 detector were recorded at an exposure of $3\ \mu s$ and calculated at 56 different delays for a maximal delay of τ_{max} of $168\ \mu s$. To match the same averaging window, the PF-32 data were averaged for every 0.125s, resulting in a sampling rate of 8 Hz.

2 Data stream

The PF-32 detector was connected to a PC via USB3.0, which supported full frame data stream to the hard drive and the g_2 data were calculated in post-processing. The second pDCS setup uses a swissSPAD3 detector, that is comprised of two sensor halves, each with 250 × 500 pixels. Each half was connected to one FPGA unit (XEM7360-K410T, OpalKelly, Portland, USA).

This study's general data processing pipeline is shown in Figure S2. The system offered two different modes of operation to obtain the g_2 data, as also explained in the main text of this publication.

3 BFI model via semi-infinite diffusion correlation equation

To derive the blood flow index (BFI) from the autocorrelation data, we used semi-infinite diffusion model that has been made publicly available by Wu. et al. (see Ref. [51] in main manuscript). This model assumes the BFI as the diffusion coefficient that causes the mean square displacement of scatteres $\langle \Delta r \rangle$, according to:

$$\langle \Delta r^2 \rangle = 6 * \alpha * BFI * \tau \tag{1}$$

This mean square displacement causes underlying fluctuations of the electric field autocorrelation function $G_1(\tau)$:

$$G_1 = \frac{3\mu_s'}{4 * \pi} * \left(\frac{e^{(-K * r_1)}}{r_1} - \frac{e^{(-K * r_2)}}{r_2} \right) \tag{2}$$

with the terms:

$K = \sqrt{3 * \mu_a * \mu_s' + \alpha * \mu_s'^2 * k_0^2 * \Delta r^2}$

$k_0 = \frac{2 * pi * n}{\lambda}$

$R = -1.440/n^2 + 0.710/n + 0.668 + 0.0636n$

$z_e = \frac{2(1+R)}{3(1-R)}$

$z_b = \frac{z_e}{\mu_s'}$

$z_0 = \frac{1}{\mu_a + \mu_s'}$

$r_1 = \sqrt{(SDS)^2 + z_0^2}$

$r_2 = \sqrt{(SDS)^2 + (z_0 + 2 * z_b)^2}$

Finally, the normalized electric field autocorrelation function $g_1(\tau)$ can be expressed as:

$$g_1 = \left| \frac{G_1}{G_1(\tau_0)} \right| \quad (3)$$

with:

$$G_1(\tau_0) = \frac{3\mu_s'}{4 * \pi} * \left(\frac{e^{(-K(\tau_0) * r_1)}}{r_1} - \frac{e^{(-K(\tau_0) * r_2)}}{r_2} \right) \quad (4)$$

and $K(\tau_0) = \sqrt{(3 * \mu_a * \mu_s')}$

A basic optical tissue properties were assumed based on literature, as displayed in table 2. These values are plausible assumptions, based on literature. The reduced scattering coefficient μ_s' of skin at 785 nm has been reported in the range of 10 cm^{-1} (see Ref. [52-53] in main manuscript) and $\mu_s' = 0.35 - 3.6\ mm^{-1}$ (see Ref. [54] in main manuscript). Here we chose $\mu_s' = 0.85 mm^{-1}$, which was used in DCS before (see Ref. [55-56] in main manuscript).

Table 2 Optical properties in semi-infinite diffusion correlation model

Parameter	Value
λ	785 nm
μ_a	0.01 mm^{-1}
μ_s'	0.85 mm^{-1}
α	1
n	1.37

The electric field autocorrelation function in Equ. 3 was inserted in the Siegert relation (see Equ. 3 in the main manuscript), where β and the BFI are optimized to match the model to the data. The initial guess for β was set to y-axis intersect of the respective g_2 curve $(g_2(\tau_0) - 1)$ with fit boundaries between 0 and 0.7. In the case of full-frame data (all data in the arm occlusion experiment and the short SDS data of the cognitive experiment), this optimization was performed by lsqcurvefit (Matlab R2021b) with the following parameters: 'TolFun' = 1e-5,'TolX' = 1e-12, 'MaxFunEvals' = 1e5, 'MaxIter' = 1e5, 'StepTolerance' = 1e-3, 'OptimalityTolerance' = 1e-5. In case that the g_2 data was calculated on-board on the FPGA, the data contained much fewer points (12) and was thus more prone to overfitting or getting stuck in local minima. Therefore, the model was optimized by particleswarm (Matlab R2021b) with the following parameters: 'SwarmSize' = 700, 'HybridFcn' = @fmincon, 'FunctionTolerance' = 1e-20, 'MaxStallIterations' = 100, 'MaxIterations' = 500) to ensure convergence of the fit.

Based on initial tests, we decided to implement an exclusion criterion to prevent misleading analysis of lower quality measurements. Therefore, we defined a cutoff of 0.03 for the model's residual and all instances above this cutoff were removed from further statistical analysis. The same procedure was applied to both detection schemes (PF-32 data at short SDS and swissSPAD3 data at long SDS), as well as to both experiments (blood flow suppression in the forearm and cognitive activity in the PFC). We pre-registered this data analysis plan (see reference [42] in main manuscript) and applied it in this study. The data of these residuals and the cutoff exclusion

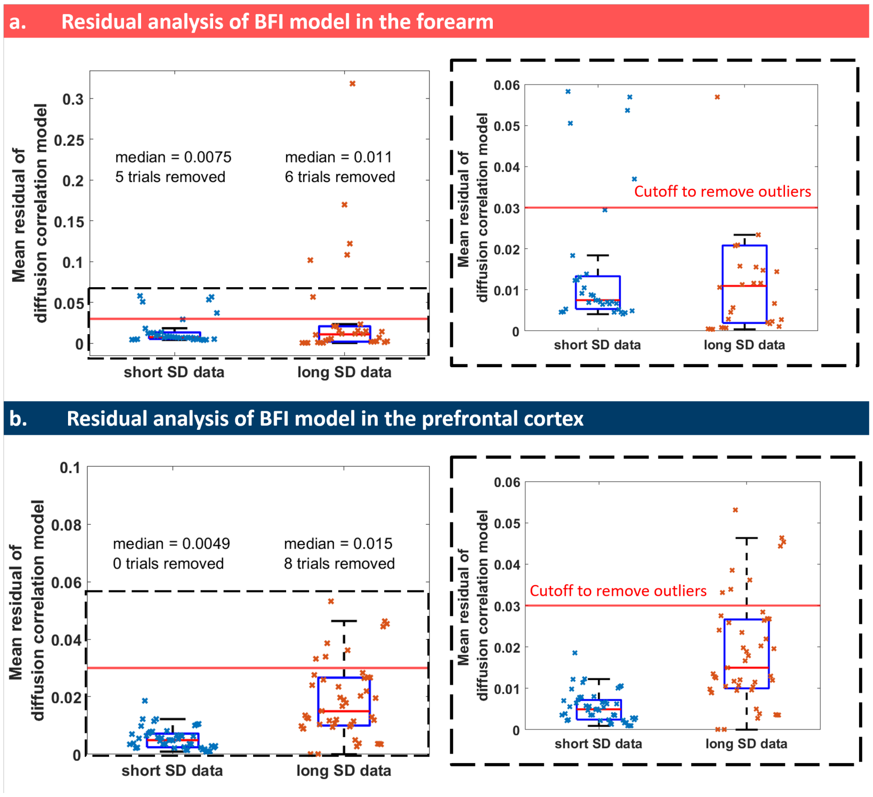

Fig S3 Residual analysis of the BFI model and cutoff value. (a) Residual distribution for the forearm experiment (0.1 s averaging window, 3 μs exposure for SDS = 4 cm, 10 μs exposure for SDS = 4 cm). (b) Residual distribution for the PFC experiment (0.125 s averaging window, 3 μs exposure for SDS = 4 cm, 3.3 μs exposure for SDS = 4 cm).

criterion are shown in Fig. S3.

In the case of the on-board calculation for g_2, with only 15 data points in each g2 curve, the autocorrelation function converged to a g_2 value of > 1 (approximated 1.1 in most cases). In these instances, this offset from the y=1 axis was subtracted from the data before fitting.

Then the same semi-infinite diffusion correlation equation was fitted to that data and optimized by particleswarm (Matlab R2021b) with the following parameters: 'SwarmSize',700, 'HybridFcn',@fmincon, 'FunctionTolerance',1e-20, 'MaxStallIterations',100, 'MaxIterations',500

4 Average speckle intensity & hot pixels

The average speckle intensity was determined to confirm if the observed difference is pDCS metric was driven or affected by changes in intensity, that might have been caused by undesired changes

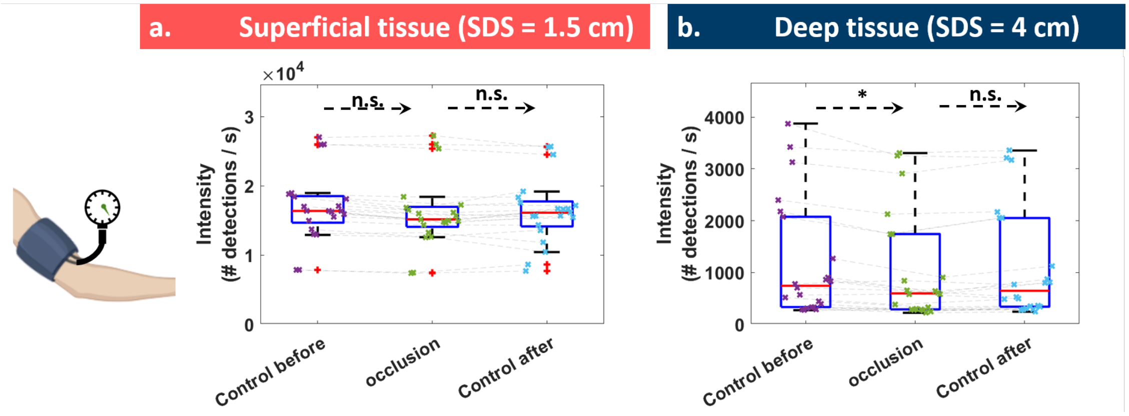

Fig S4 Average speckle intensity in each SPAD pixel. (a) Superficial signal at 1.5 cm source-detector separation. (b) Deep signal at 4 cm source-detector separation. All boxplots show the 25th and 75th percentile as boxes. Significance levels are indicated as $^{*}p < 0.05,^{**} p < 0.01,^{***} p < 0.001$.

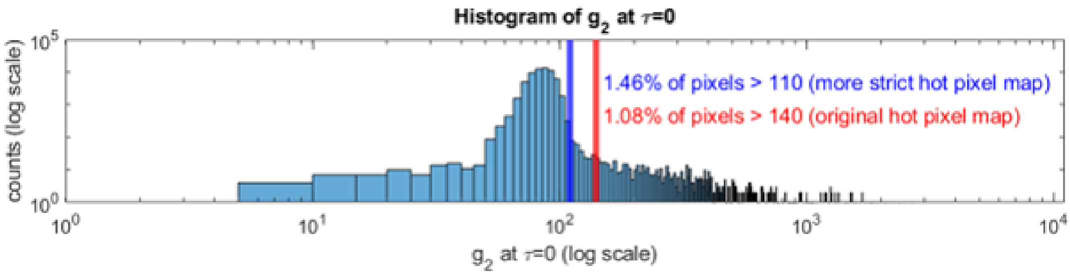

Fig S5 Histogram of $g_2(\tau = 0)$ from a dark measurement. This measurement of g_2 at $\tau = 0$ was recorded with the on-board FPGA autocorrelation calculation and to determine hot pixels for this measurement. The red line indicates that cutoff value for the hot pixel map that was used in this study, which exlcuded about 1% of pixels above the value of $g_2(\tau = 0)$ = 140.

in measurement fidelity or by systematic changes in absorption properties of the tissue under investigation. This intensity was calculated as the average number of photon detection events across all pixels in the frame at a given time point.

Fig. S5 shows the histogram of $g_2(\tau = 0)$ values per pixel from a dark measurement, which was used to determine hot pixels.

5 Initial slope of autocorrelation curve

Besides the established BFI, we tested the initial g_2 difference between the first two τ values:

$$\Delta g_{2,init} \; = \; g_2(\tau_1) - g_2(\tau_2) \tag{5}$$

The rationale for this metric is that it carries information on changes in speckle contrast, similar to (r)BFI, but focusing solely on the two earliest delay values at highest blood flow sensitivity and SNR. Since the g_2 curve decayed relatively fast at this large SDS, it was tested for blood flow sensitivity. This initial g_2 difference was sampled at the same rate as the BFI (8 Hz for the cognitive activation experiment and 10 Hz for the suppression experiment). For the statistical analysis, this initial g_2 slope values were normalized to 100 % of the median of the first control measurement. The data from each of the two detectors was analyzed separately so that the short source-detector separation (SDS = 1.5 cm) could serve as an in-built control measurement for the deep PDCS configuration (SDS = 4 cm).

In addition to the more rigorous and well-established BFI (Fig. 3 & 5 in the main paper), we tested the initial g_2 slope as blood flow metric (Fig. S6 & S7). This metric follows the same general trend in the group statistics as the BFI described above - albeit at a different magnitude. For the superficial tissue measurement in the forearm, this metric shows a highly significant decrease of 46 % and 63 % when compared to the control before or after, respectively. For the deep tissue PDCS measurement in the same experiment, the average decrease of this metric is 62 % and 63 %. Again, these results were statistically significant in all conditions and at both measurement depths ($p < 0.001$).

In the cognitive experiment, the initial g_2 slope (Fig. S7) shows a similar trend as the BFI.

Data from the superficial PDCS configuration shows statistical significance of this metric, when comparing the n-back test to the control before with an average increase of 20 % ($p < 0.001$). However, no significant change was detected when comparing the test to the control after ($p > 0.05$). In the deep PDCS configuration, the data show a statistically significant increase in both conditions ($p < 0.001$). The average change is 15 % and 12 %, when compared to the control before or after, respectively (Fig. S7 c1).

Although this trend is apparent and statistically significant, the magnitude of the change in the initial g_2 slope of >60 % for the arm or >15 % in the PFC should *not* be mistaken for an equivalently large difference in flow. The magnitude of this change is not linearly proportional to actual blood flow changes, since it might be affected by several other parameters, like changes in absorption (e.g., due to increased hemoglobin volume), fluctuations β and others. In order to investigate if this difference was indeed driven by an increased hemoglobin volume, we measured the average speckle intensity, which would indicate absorption changes, similar to a conventional NIRS measurement. As seen in the supplementary material, this intensity metric did not indicate a distinct difference of statistical significance, when comparing cognitive activation with the control cases.

6 Pulsatility analysis

As shown in the methods section of the main paper, we were able to identify systolic peaks (SP), diastolic end points (DE), diastolic peaks (DP) and diastolic notches (DN) in the BFI traces. In addition to the pulsatility index, we also calculated the resistance index (RI) and the notch index

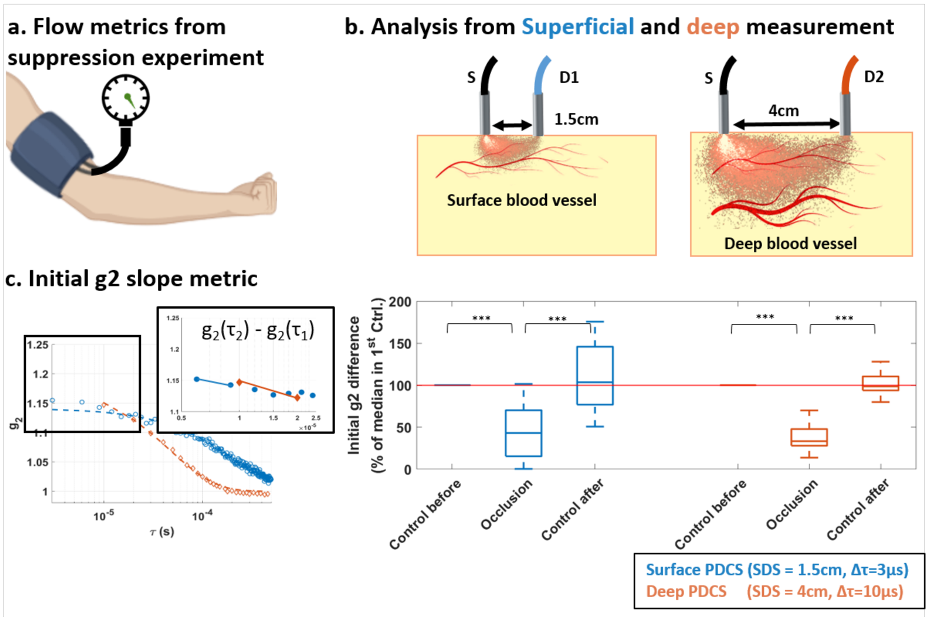

Fig S6 Initial g_2 slope metric (a) of muscle blood flow (MBF) measurements via PDCS during blood flow suppression via pressurized cuff. (c) The median of the initial g_2 slope was derived for each individual trial and results of all trials were compared between the three conditions (control before, test, control after) and for both detection schemes (superficial PDCS at SDS=1.5 cm in blue and deep PDCS at SDS=4cm in orange). All results show a statistically significant decrease during blood flow suppression ($*p < 0.05$). All boxplots show the 25th and 75th percentile as boxes. Significance levels are indicated as $^*p < 0.05,^{**} p < 0.01,^{***} p < 0.001$. Data include 33 independent trials from 11 subjects.

(NI) were calculated as, based on these definitions (see Ref. [59] in main manuscript):

$$RI = \frac{\langle BFI \rangle_{SP} - \langle BFI \rangle_{DE}}{\langle BFI \rangle_{SP}} \tag{6}$$

$$NI = \frac{\langle BFI \rangle_{DP} - \langle BFI \rangle_{DN}}{\langle BFI \rangle} \tag{7}$$

Where $\langle BFI \rangle$ is the average BFI of the entire trial, while $\langle BFI \rangle_{SP}$ denotes the respective average across all detected systolic peaks, $\langle BFI \rangle_{DE}$ across all diastolic end points, $\langle BFI \rangle_{DP}$ across all diastolic peaks and $\langle BFI \rangle_{DN}$ across all diastolic notches. The results are displayed in Fig. S8

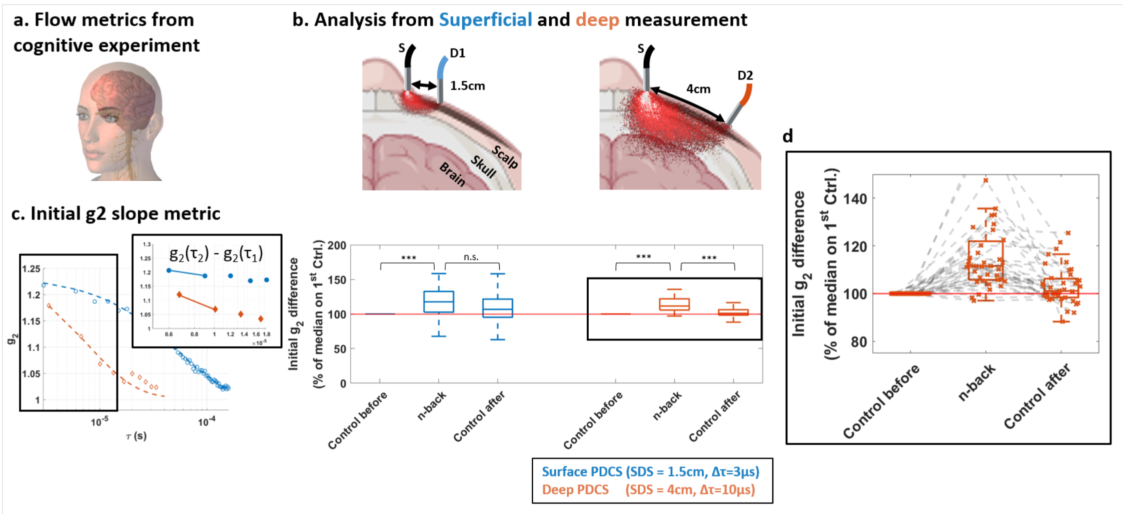

Fig S7 Initial g_2 slope metric (a) of cerebral blood flow (CBF) measurements via PDCS during functional activation task. (c) The median of the initial g_2 slope was derived for each individual trial and results of all trials were compared between the three conditions (control before, n-back, control after) and for both detection schemes (superficial PDCS at SDS=1.5 cm in blue and deep PDCS at SDS=4cm in orange). Only the deep PDCS configuration shows a statistically significant increase during the cognitive task ($*p < 0.05$), indicating cerebral sensitivity. (c1) shows a re-scaled version of the group differences from the deep tissue layer (at SDS = 4 cm). All boxplots show the 25th and 75th percentile as boxes. Significance levels are indicated as $^*p < 0.05,^{**}p < 0.01,^{***}p < 0.001$. Data include 39 independent trials from 15 subjects.

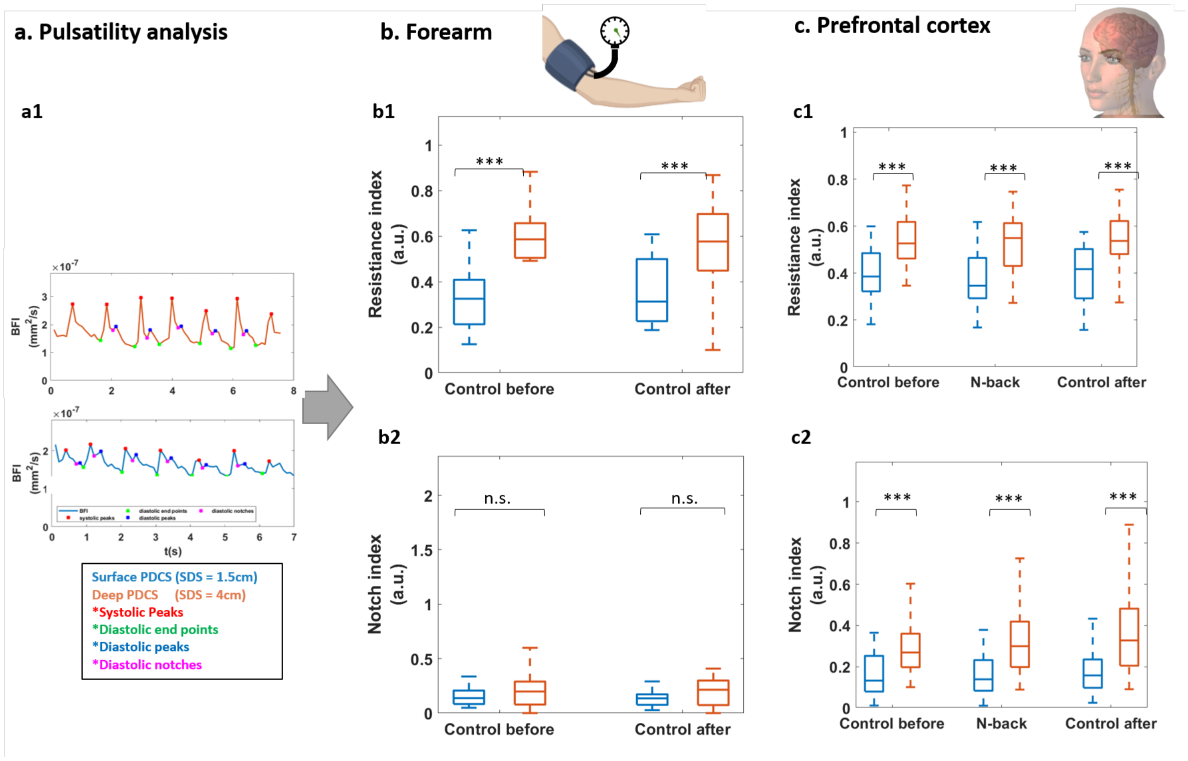

Fig S8 Resistance index and notch index for the different experiments.

7 Noise analysis

In addition to the noise analysis of the full array (see main paper), we also analyzed the noise as a function of τ and different pixel counts M_{pixel} to evaluate if the noise reduction follows the expected $\sqrt{M_{pixel}}$ trend. Therefore, 25 different subsets of exponentially increasing pixel counts between 1 the maximal pixel number were randomly selected using Matlab's 'randperm' function. The g_2 curve was averaged across all pixels in the respective subset and the noise $N(\tau, M_{pixel})$ was calculated as the standard deviation across n systolic peaks or diastolic end points:

$$N(\tau, M_{pixel}) = std(\langle g_2(\tau) \rangle_{M_{pixel}})_n \tag{8}$$

where $g_2(\tau)$ is the autocorrelation function, averaged across the M_{pixel} pixels in the subset and std() is the standard deviation across n data points under similar conditions (i.e., systolic peaks or diastolic end points respectively).

This same process was repeated in k-fold cross validation loop to account for the randomness of selected pixel subsets. Mean and standard deviation of the noise from this k-fold validation were then plotted against the number of pixels in each subset, as seen in Fig. S9 & S10. In case of the 1,024 pixel array at 1.5 cm, the procedure was repeated 15 times (k = 15) in both experiments. In the case of the larger array at 4 cm, the procedure was repeated 1000 times (k = 1000) and the g_2 was normalized to its y-axis offset to account for the variation in β.

The previous work on optical phantoms further showed a 473X boost in SNR and confirmed the expected $\sqrt{N_{pixels}}$ behavior (see Ref. [40] in main manuscript). Our *in vivo* results from this pixel-level noise analysis (Fig. S9 & S10) also mostly follows this $\sqrt{N_{pixels}}$ rule, albeit at slightly lower maximal boost, as it includes additional biological variations other potential sources

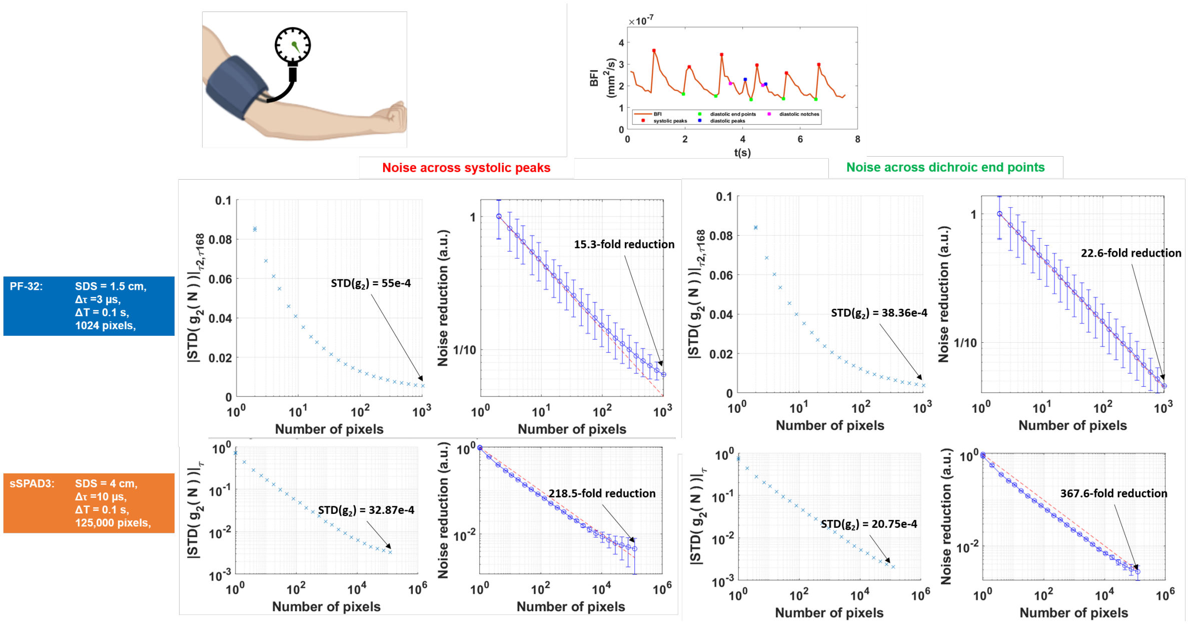

Fig S9 Noise analysis from different subsets of increasing pixel counts from one exemplary trial from the blood flow suppression experiment in the forearm. The noise was calculated as the standard deviation between systolic peaks (left) or between diastolic end points (right) for both detector configurations. This noise was averaged across all delay values τ and plotted against the number of pixels in each subset in absolute values (left column in each case) as well as in relative values, normalized to the subset that contain one random pixel (right column in each case). This procedure was repeated several times in a k-fold cross validation loop to account for the randomness of selecting pixel subsets.

for noise of *in vivo* experiments, that are usually not present during phantom studies. This shortcoming can be observed in maximal noise reduction can be observed in all possible conditions (in systolic peaks and diastolic end points, in both detection schemes and in both experiments). Thus, it is probably attributed to the fact, this this overall noise analysis includes not only the expected technical noise, but also all other deviations between subsequent BFI values, related to systematic variations, biological variability, natural variation between different peaks and notches in the same BFI trace etc.

Nevertheless, our results generally show that the absolute noise level at large SDS with the larger array remains on the same level as that of the smaller array at shorter SDS. In a previous experiment on an idealized and controllable optical phantom, the noise was calculated across 120 consecutive measurements to be $2.6x10^{-4}$ at the single initial delay value of $\tau = 10.81\ \mu s$ and

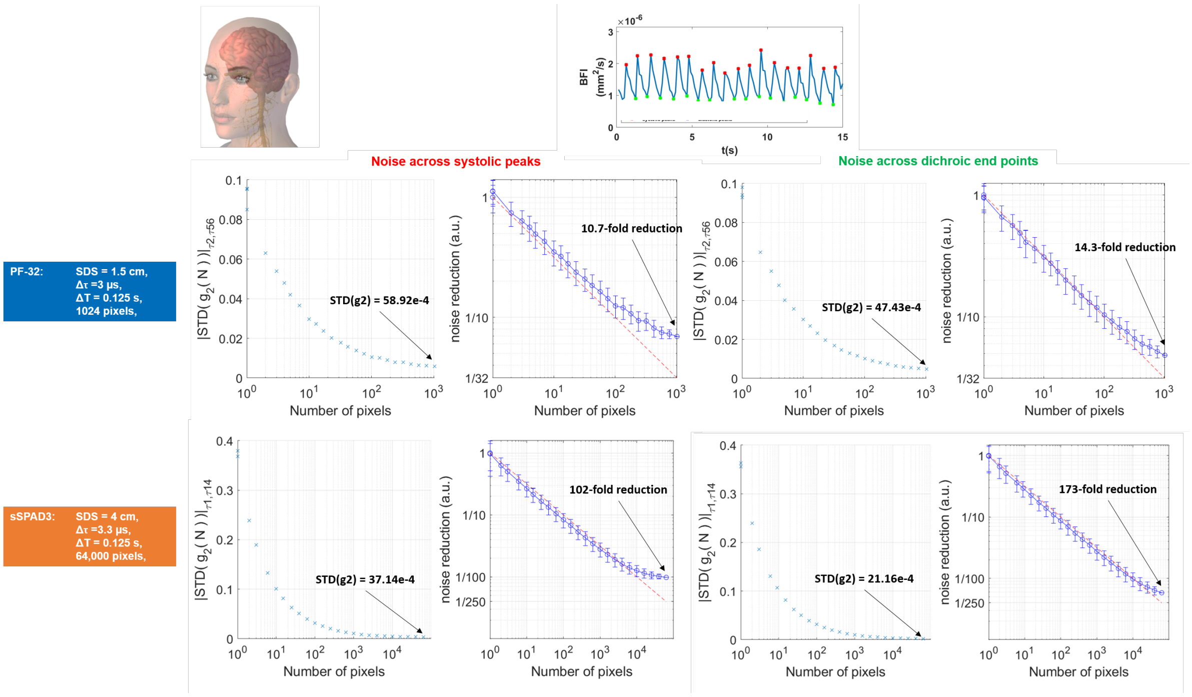

Fig S10 Noise analysis from different subsets of increasing pixel counts from one exemplary trial from the blood flow activation experiment in the PFC. The noise was calculated as the standard deviation between systolic peaks (left) or between diastolic end points (right) for both detector configurations. This noise was averaged across all delay values τ and plotted against the number of pixels in each subset in absolute values (left column in each case) as well as in relative values, normalized to the subset that contain one random pixel (right column in each case). This procedure was repeated several times in a k-fold cross validation loop to account for the randomness of selecting pixel subsets.

using all 250,000 pixels (see Ref. [40] in main manuscript). In our experiments, using fewer pixels and including biological noise, the average noise across all delay times in only 5-20 consecutive systolic peaks or diastolic endpoints was mostly within the range of $30x10^{-4} - 270x10^{-4}$.
